# Development and Validation of a Prognostic Survival Model With Patient-Reported Outcomes for Patients With Cancer

**DOI:** 10.1001/jamanetworkopen.2020.1768

**Published:** 2020-04-01

**Authors:** Hsien Seow, Peter Tanuseputro, Lisa Barbera, Craig Earle, Dawn Guthrie, Sarina Isenberg, Rosalyn Juergens, Jeffrey Myers, Melissa Brouwers, Rinku Sutradhar

**Affiliations:** 1 Department of Oncology, McMaster University, Hamilton, Ontario, Canada; 2 Institute for Clinical Evaluative Sciences, Toronto, Ontario, Canada; 3 Division of Palliative Care, Department of Medicine, University of Ottawa, Ottawa, Ontario, Canada; 4 Ottawa Hospital Research Institute, Ottawa, Ontario, Canada; 5 Department of Oncology, University of Calgary, Calgary, Alberta, Canada; 6 Tom Baker Cancer Centre, Alberta Health Services, Calgary, Alberta, Canada; 7 Canadian Partnership Against Cancer, Toronto, Ontario, Canada; 8 Department of Kinesiology and Physical Education, Department of Health Sciences, Wilfrid Laurier University, Waterloo, Ontario, Canada; 9 Temmy Latner Centre for Palliative Care, Lunenfeld Tanenbaum Research Institute, Sinai Health System, Toronto, Ontario, Canada; 10 Division of Palliative Care, Department of Family and Community Medicine, University of Toronto, Toronto, Ontario, Canada; 11 University of Ottawa School of Epidemiology and Public Health, Ottawa, Ontario, Canada; 12 Dalla Lana School of Public Health, University of Toronto, Toronto, Ontario, Canada

## Abstract

**Importance:**

Existing prognostic cancer tools include biological and laboratory variables. However, patients often do not know this information, preventing them from using the tools and understanding their prognosis.

**Objective:**

To develop and validate a prognostic survival model for all cancer types that incorporates information on symptoms and performance status over time.

**Design, Setting, and Participants:**

This is a retrospective, population-based, prognostic study of data from patients diagnosed with cancer from January 1, 2008, to December 31, 2015, in Ontario, Canada. Patients were randomly selected for model derivation (60%) and validation (40%). The derivation cohort was used to develop a multivariable Cox proportional hazards regression model with baseline characteristics under a backward stepwise variable selection process to predict the risk of mortality as a function of time. Covariates included demographic characteristics, clinical information, symptoms and performance status, and health care use. Model performance was assessed on the validation cohort by C statistics and calibration plots. Data analysis was performed from February 6, 2018, to November 6, 2019.

**Main Outcomes and Measures:**

Time to death from diagnosis (year 0) recalculated at each of 4 annual survivor marks after diagnosis (up to year 4).

**Results:**

A total of 255 494 patients diagnosed with cancer were identified (135 699 [53.1%] female; median age, 65 years [interquartile range, 55-73 years]). The cohort decreased to 217 055, 184 822, 143 649, and 109 569 patients for each of the 4 years after diagnosis. In the derivation cohort year 0, and the most common cancers were breast (30 855 [20.1%]), lung (19 111 [12.5%]), and prostate (18 404 [12.0%]). A total of 47 614 (31.1%) had stage III or IV disease. The mean (SD) time to death in year 0 was 567 (715) days. After backward stepwise selection in year 0, the following factors were associated with increased risk of death by more than 10%: being hospitalized; having congestive heart failure, chronic obstructive pulmonary disease, or dementia; having moderate to high pain; having worse well-being; having functional status in the transitional or end-of-life phase; having any problems with appetite; receiving end-of-life home care; and living in a nursing home. Model discrimination was high for all models (C statistic: 0.902 [year 0], 0.912 [year 1], 0.912 [year 2], 0.909 [year 3], and 0.908 [year 4]).

**Conclusions and Relevance:**

The model accurately predicted changing cancer survival risk over time using clinical, symptom, and performance status data and appears to have the potential to be a useful prognostic tool that can be completed by patients. This knowledge may support earlier integration of palliative care.

## Key Points

**Question:**

Is it possible to develop a risk prediction model of survival for patients with cancer that incorporates patient-reported outcomes over time?

**Findings:**

In this prognostic study of data from 255 494 patients with cancer, the mean) time to death from diagnosis was 567 days. The model found that the following factors were associated with increased risk of death by more than 10%: being hospitalized; having congestive heart failure, chronic obstructive pulmonary disease, or dementia; having moderate to high pain; having worse well-being; having functional status in the transitional or end-of-life phase; having any problems with appetite; receiving end-of-life home care; and living in a nursing home.

**Meaning:**

Patients and families may be more informed when making decisions, such as about treatments or palliative care, if they can easily calculate prognostic information.

## Teaser

This prognostic study describes a prognostic survival model for all cancer types that incorporates information on symptoms and performance status over time.

## Introduction

Several randomized clinical trials demonstrated the benefits of early integration of palliative care (ie, at diagnosis) with active cancer treatment, such as improved quality of life and symptom control.^1,2,3^ This evidence led the American Society of Clinical Oncology to endorse the provision of early palliative care concurrently with standard oncologic care.^4^ Although evidence indicates the positive effect of palliative care integration at the time of diagnosis, patients generally receive palliative care close to death or not at all. Data from the US^5^ indicate that palliative care was accessed in 45% of all deaths for a median of 17 days before death. An increasing body of research has focused on developing prognostic tools, particularly online tools, to help practitioners predict death in patients with cancer. A systematic review identified 22 online prognostic tools that addressed 89 different cancers.^6^ However, prognostic tools largely fail to integrate palliative care earlier in the disease trajectory for several reasons.

First, most tools were designed for use by oncologists. However, even with prognostic tools, oncologists often struggle with when to discuss prognosis and palliative care because they do not want to take away patient hope, and cancer advancements have increased treatment options and clinical trials.^7,8^ When practitioners discuss prognosis, research shows their predictions are overly optimistic.^9,10^ Second, existing tools have limitations in their usefulness as the disease progresses. Many of the tools predict death from diagnosis but do not account for changes over time, such as in treatment plan or health services use. Most tools also do not incorporate patient-reported outcomes, such as performance status or symptom burden, which have clinical and statistical prognostic value across multiple cancers.^11,12^ Third, patients face barriers to use the tools directly. Systematic reviews^13,14^ have found that many prognostic tools require biological and laboratory variables, such as cancer antigen levels, elevated C-reactive protein level, and leukocytosis, which are not typically known by patients. This requirement prevents patients from obtaining prognostic information that could help them initiate discussions about palliative care.

In this study, we aimed to develop and validate a prognostic model to predict survival in patients with cancer. To address prior limitations, the model uses easily known clinical information and incorporates patient-reported outcomes (ie, common symptoms and performance status) over time using unique databases available in Ontario, Canada.^15,16^ Thus, the model can be completed by patients and families. We named the model PROVIEW by blending the goal to help patients preview the future to be proactive. PROVIEW aims to provide changing survival predictions as the disease progresses over time, which could support discussions about integrating palliative care even alongside disease-modifying therapies.

## Methods

### Study Design and Population

We performed a population-based, retrospective prognostic study of data from adults diagnosed with cancer, as confirmed by the provincial cancer registry in Ontario, Canada, from January 1, 2008, to December 31, 2015. Data analysis was performed from February 6, 2018, to November 6, 2019. The study was reviewed by Hamilton Integrated Research Ethics Board and deemed exempt because it was a deidentified, secondary data analysis. This study followed the Transparent Reporting of a Multivariable Prediction Model for Individual Prognosis or Diagnosis (TRIPOD) reporting guideline.^17^

### Data Sources

We used the following linked administrative databases (and corresponding covariates): (1) Ontario Cancer Registry (cancer type, diagnosis date, and stage); (2) Vital Statistics (age, sex, and date of death); (3) Statistics Canada (rurality, income quintile, and region); (4) Activity Level Reporting (chemotherapy and radiation treatment); (5) Discharge Abstract Database (hospitalization dates, diagnoses, surgery for cancer, and comorbidity); (6) National Acute Care Registry System (emergency department visits and reasons); (7) physician billing (physician visits and billing codes); (8) Home Care database (nursing and personal support); (9) Symptom Management database (symptoms and performance status); and (10) interRAI database (performance status and symptoms).

The 2 databases that contain population-based performance status and symptom data are the Symptom Management and interRAI databases. The Symptom Management database began in 2007 when Cancer Care Ontario mandated the systematic screening of outpatients with cancer for symptoms using the Edmonton Symptom Assessment System (ESAS) and for performance status using the Palliative Performance Scale (PPS).^15^ Every patient being treated at a cancer center is eligible to complete the ESAS and PPS, both of which are validated tools in populations with cancer.^18,19^ The monthly provincial screening completion rate is 56%.^20^ The ESAS asks patients to self-report the severity of 9 symptoms (ie, pain, depression, well-being, shortness of breath, anxiety, nausea, tiredness, drowsiness, and appetite) on a scale of 0 (symptom absent) to 10 (most severe), whereas the PPS describes a patient’s performance status based on a patient’s level of ambulation, level of activity, and ability to perform self-care. The PPS is scored from 0 to 100 (in 10-point increments), with 80 to 100 indicating stable, 40 to 70 indicating transitional, 10 to 30 indicating end of life, and 0 indicating dead. The PPS is completed by the practitioner during the patient’s visit. In 2013, Ontario also began collecting functional scores using a patient-completed Eastern Cooperative Oncology Group score, which is comparable and highly correlated with the physician-reported PPS.^21,22^

The interRAI database began in 2002, when Ontario mandated the use of the Resident Assessment Instrument for Home Care, a standardized tool for patients receiving publicly funded home care services for an expected 60 days or more. The assessment is akin to the Minimum Data Set used internationally and is valid and reliable.^23,24,25^ Seventy percent of patients with cancer use home care in the last year of life.^26^ The assessment collects quality-of-life data for approximately 300 unique items that measure domains, such as the presence of moderate to severe pain or depression, presence of caregiver living in patient’s home, and performance status via the health instability CHESS scale (change in decision-making, change in activities of daily living status, and end-stage disease).^27^ The assessment is completed by the case manager at intake and reassessed at least every 6 months.

### Outcome

The primary outcome was time to death (days) per date of death in the Vital Statistics database. The initial index date for each patient was the date of diagnosis. Because covariates and treatments may change over time, we also aimed to predict conditional survival probabilities; thus, prediction models were redeveloped by moving the index date to the 1-, 2-, 3-, and 4-year survival marks. Only patients who were alive at those marks contributed to each corresponding conditional analysis. All covariates were recalculated at each new index date to avoid incorporating time-varying covariates into the regression model because predictions are meant to be based on information known only at the current time.

### Covariates

Each model included the following baseline covariates: demographic characteristics (age at diagnosis, sex, caregiver living with the patient [yes or no], and lives within 50 km of a cancer center [yes or no]); clinical data (diagnosis date, cancer type, cancer stage, presence of 1 of 13 other chronic diseases as determined by validated algorithms,^28,29^ type of chemotherapy [publicly funded oral drugs, immunotherapy, and systemic agents], receipt of radiation treatment [yes or no], and/or cancer surgery [yes or no] in the past [from diagnosis up to 3 months previously] and recently [within the past 3 months]); patient-reported outcomes (performance status and 9 symptom scores within 3 months of index date); and health care use within 3 months of the index date (prior hospitalization, hospitalizations for palliative care [including palliative care consultation], living in long-term care, receipt of end-of-life home care services, having a regular family physician, and received physician home visit).

### Statistical Analysis

For the primary outcome of time to death, prediction methods for time-to-event data were implemented starting from diagnosis and then reimplemented at each of the 4 yearly survival marks after diagnosis. Each derived prediction model followed the below steps.

#### Developing the Prediction Model

We randomly selected 60% of eligible patients for model derivation and used the other 40% for validation. To ensure random sampling, we assessed and compared the distribution of baseline characteristics between the derivation and validation cohorts. Using the derivation cohort, we used a multivariable Cox proportional hazards regression model with baseline (time-fixed) characteristics to predict the hazard of mortality as a function of time. A priori, we created a multivariable model that consisted of all potential variables mentioned above. We then used the backward stepwise selection procedure for variable selection with a liberal 2-sided *P* < .15 as the retention criteria.^30^ The proportional hazards assumption was assessed by including interactions with time and each covariate into the model. We centered continuous covariates, such as age, and explored linear and quadratic terms. Missing data from patient-reported categorical variables were handled by creating an additional missing category for that variable. Most of the missing data were attributable to patients not completing an ESAS, although occasionally patients skip certain questions on the ESAS. Because there was no obvious missing pattern, we elected to create a missing category rather than to impute or remove these patients from the analysis. Interactions between cancer type and stage were also incorporated with a goal of achieving maximal discriminative ability within the derivation cohort,^31,32^ as determined by the concordance index.^33^

#### Validating the Prediction Model

After the final regression model was established, the 1-year predicted probability of death was calculated for each patient in the validation cohort based on their specific covariate values, the estimates of the regression variables from step 1, and the estimate of the baseline survival function from step 1.^34^ Calibration (how close the model-estimated risk is to the observed risk) was examined by grouping patients into deciles of model-estimated 1-year risk of death. We then reviewed the plot of the observed against the predicted 1-year probabilities of death for patients in each decile.^35^

We measured the model’s discriminative ability (ability to distinguish between patients who died from those who did not die) via a concordance index (C index).^36,37^ Concordance for survival data was calculated as the proportion of pairs in which the patient who died had a higher predicted probability than the patient who did not die. All analyses were conducted using the statistical software R, version 2.15 (R Project for Statistical Computing) and SAS, version 9.3 (SAS Institute Inc).

## Results

**Figure 1.**
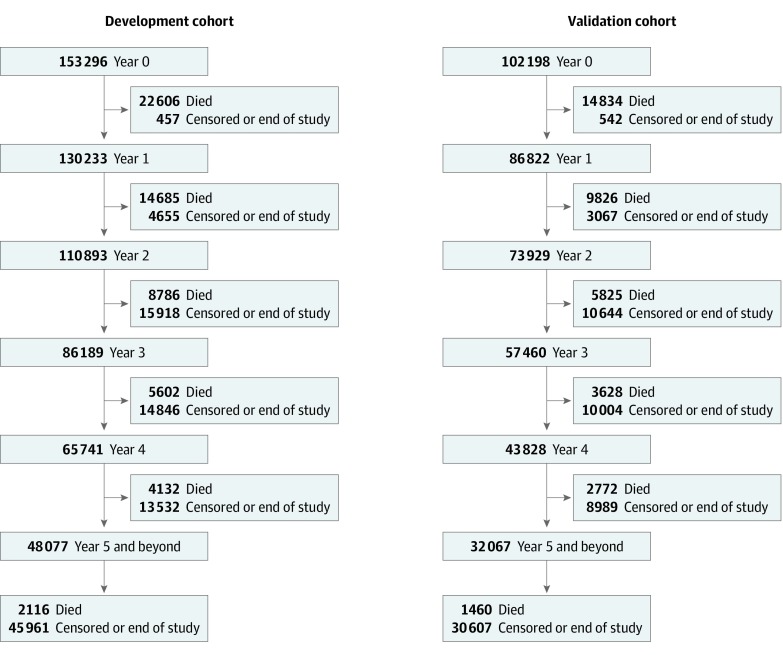
Flow Diagram of the Development and Validation Cohorts

We identified 255 494 patients (135 699 [53.1%] female; median age, 65 years [interquartile range, 55-73 years]) diagnosed with cancer during 2008 to 2015. Because we repeated the derivation and validation process each year up to 4 years after diagnosis conditional on survival, the total cohort decreased to 217 055 in year 1, 184 822 in year 2, 143 649 in year 3, and 109 569 in year 4 (Figure 1). We randomly split each total cohort into derivation (60%) and validation cohorts (40%). Characteristics between the derivation and validation cohort were nearly identical in the diagnosis year (year 0).(Table 1). In the derivation cohort year 0, the most common cancers were breast (30 855 [20.1%]), lung (19 111 [12.5%]), prostate (18 404 [12.0%]), and colorectal (16 776 [10.9%]). A total of 47 614 (31.1%) of the cohort had stage III or IV disease, 66 958 (43.7%) had stage I or II disease, and 38 724 (25.2%) had unknown stage in the registry. Within the first 3 months of diagnosis, 71 479 (46.6%) had cancer-related surgery, 41 486 (27.1%) received chemotherapy, and 37 581 (24.5%) received radiation therapy. Although half of the patients did not have a performance status recorded within 3 months of diagnosis, 13 320 (8.7%) were in the transitional stage and 1 709 (1.1%) were at end of life. A total of 23 818 (15.5%) of the cohort had moderate to high pain, 43 879 (28.6%) had no pain, and 62 049 (40.5%) had missing values. Within 3 months of diagnosis, 10 172 (6.6%) were hospitalized for palliative care intent, and 9 038 (5.9%) received end-of-life home care services. The main difference between the year 4 and year 0 derivation and validation cohorts was that fewer patients were still receiving treatment in year 4 (eTable 1, eTable 2, eFigure 1, and eFigure 2 in the Supplement include all variables across all years and additional analyses).

**Table 1.** Baseline Characteristics of the Study Cohort at Years 0 and 4^a^

Characteristic	Year 0		Year 4	
	Derivation cohort (n = 153 296)	Validation cohort (n = 102 198)	Analysis cohort (n = 65 741)	Validation cohort (n = 43 828)
Age at diagnosis, median (IQR), y	65 (55-73)	65 (55-73)	66 (57-75)	67 (57-75)
Female	81 568 (53.2)	54 131 (53.0)	36 213 (55.1)	24 328 (55.5)
Cancer type^b^				
Breast	30 855 (20.1)	20 719 (20.3)	17 483 (26.6)	11 618 (26.5)
Colorectal	16 776 (10.9)	11 142 (10.9)	7429 (11.3)	4928 (11.2)
Lung	19 111 (12.5)	12 604 (12.3)	2802 (4.3)	1904 (4.3)
Prostate	18 404 (12.0)	12 500 (12.2)	11 888 (18.1)	7820 (17.8)
Cancer stage^c^				
I	32 505 (21.2)	21 557 (21.1)	18 845 (28.7)	12 587 (28.7)
II	34 453 (22.5)	23 179 (22.7)	19 704 (30.0)	13 144 (30.0)
III	24 624 (16.1)	16 581 (16.2)	10 162 (15.5)	6739 (15.4)
IV	22 990 (15.0)	15 277 (14.9)	3415 (5.2)	2176 (5.0)
Radiation in past 3 mo	37 581 (24.5)	25 156 (24.6)	1834 (2.8)	1159 (2.6)
Chemotherapy in past 3 mo	41 486 (27.1)	27 669 (27.1)	6457 (9.8)	4291 (9.8)
Surgery for cancer in past 3 mo	71 479 (46.6)	47 627 (46.6)	2857 (4.3)	1907 (4.4)
Distance from regional cancer center ≤50 km	121 902 (79.5)	81 800 (80.0)	52 582 (80.0)	34 961 (79.8)
Patient hospitalized in the past 3 mo	9682 (6.3)	6369 (6.2)	6212 (9.4)	4166 (9.5)
Chronic disease^d^				
CHF	8470 (5.5)	5695 (5.6)	4566 (6.9)	2973 (6.8)
COPD	13 567 (8.9)	9048 (8.9)	5724 (8.7)	3830 (8.7)
Dementia	2543 (1.7)	1613 (1.6)	1757 (2.7)	1158 (2.6)
Functional score at index within 3 mo^c^^,^^e^				
100	34 314 (22.4)	23 316 (22.8)	10 605 (16.1)	7222 (16.5)
80-90	21 596 (14.1)	14 429 (14.1)	6099 (9.3)	4187 (9.6)
60-70	8427 (5.5)	5487 (5.4)	1749 (2.7)	1157 (2.6)
40-50	4893 (3.2)	3216 (3.1)	977 (1.5)	627 (1.4)
10-30	1709 (1.1)	1101 (1.1)	299 (0.5)	201 (0.5)
Pain score at index within 3 mo^c^				
None	43 879 (28.6)	29 311 (28.7)	12 944 (19.7)	8703 (19.9)
Low	23 550 (15.4)	16 078 (15.7)	5686 (8.6)	3800 (8.7)
Moderate	15 705 (10.2)	10 374 (10.2)	2753 (4.2)	1964 (4.5)
High	8113 (5.3)	5386 (5.3)	1380 (2.1)	888 (2.0)
Well-being score at index within 3 mo^c^				
None	23 898 (15.6)	16 078 (15.7)	8417 (12.8)	5777 (13.2)
Low	35 305 (23.0)	23 905 (23.4)	8743 (13.3)	5939 (13.6)
Moderate	23 941 (15.6)	15 868 (15.5)	4073 (6.2)	2711 (6.2)
High	10 617 (6.9)	6966 (6.8)	1588 (2.4)	1003 (2.3)
Appetite within 3 mo^c^				
No problems, best appetite	40 624 (26.5)	27 448 (26.9)	14 525 (22.1)	9910 (22.6)
Low	17 402 (11.4)	11 691 (11.4)	3722 (5.7)	2473 (5.6)
Moderate	14 364 (9.4)	9421 (9.2)	1890 (2.9)	1261 (2.9)
High, worst appetite	9874 (6.4)	6516 (6.4)	906 (1.4)	561 (1.3)
Hospitalized for palliative care	10 172 (6.6)	6581 (6.4)	1662 (2.5)	1076 (2.5)
Resides in long-term care facility	1721 (1.1)	1096 (1.1)	1040 (1.6)	690 (1.6)
Received home care	9038 (5.9)	5951 (5.8)	1507 (2.3)	1029 (2.3)

Abbreviations: CHF, congestive heart failure; COPD, chronic obstructive pulmonary disease; IQR, interquartile range.

^a^ Data are presented as number (percentage) of patients unless otherwise indicated.

^b^ Other cancer disease sites were other genitourinary, other gastrointestinal, hematologic, head and neck, gynecologic, and other sites.

^c^ Percentages do not total 100% because the missing category is not shown.

^d^ Other chronic diseases measured but not reported were acute myocardial infarction, arrythmia, asthma, coronary heart disease, diabetes, hypertension, inflammatory bowel disease, mood disorder, osteoarthritis, osteoporosis, renal disease, rheumatoid arthritis, and stroke; mental health hospital admission was also measured but not reported.

^e^ Functional score ranges from 0 to 100 (in 10-point increments), with 80 to 100 indicating stable, 40 to 70 indicating transitional, 10 to 30 indicating end of life, and 0 indicating dead.

After backward stepwise selection, each yearly survival model had a different set of variables included in the final prediction model (Table 2). In the year 0 model, the following factors were associated with increased instantaneous risk of death by more than 10%: having lung cancer; having worse than stage I disease; being hospitalized for any reason; and, especially if the main reason was for palliative care, having congestive heart failure, chronic obstructive pulmonary disease, or dementia; having moderate or high pain; having worse well-being; having a performance status in the transitional or end-of-life phase; having any problems with appetite; receiving end-of-life home care; and living in a nursing home.

**Table 2.** Final Time-to-Death Model for Developmental Cohort Following Backward Elimination^a^

Variable	HR (95% CI)	
	Year 0	Year 4
Age at index date, centered	1.03 (1.03-1.03)	1.03 (1.03-1.04)
Female	0.87 (0.85-0.89)	0.82 (0.78-0.87)
Cancer type^b^		
Lung	1 [Reference]	1 [Reference]
Breast	0.33 (0.31-0.34)	0.54 (0.49-0.60)
Colorectal	0.51 (0.50-0.53)	0.73 (0.66-0.81)
Prostate	0.20 (0.19-0.21)	0.45 (0.41-0.51)
Cancer stage^b^		
I	1 [Reference]	1[Reference]
II	1.64 (1.57-1.70)	1.17 (1.08-1.26)
III	2.70 (2.61-2.80)	1.45 (1.34-1.58)
IV	5.05 (4.87-5.23)	1.98 (1.80-2.17)
Admitted to hospital in past 3 mo	1.17 (1.14-1.20)	1.94 (1.83-2.07)
Chronic disease		
CHF	1.21 (1.17-1.25)	1.41 (1.32-1.51)
COPD	1.19 (1.16-1.22)	1.38 (1.29-1.47)
Dementia	1.17 (1.11-1.23)	1.63 (1.48-1.79)
Radiation in past 3 mo	1.12 (1.09-1.14)	1.48 (1.36-1.60)
Chemotherapy in past 3 mo	1.06 (1.04-1.08)	2.18 (2.03-2.33)
Surgery for cancer in past 3 mo	0.77 (0.75-0.78)	NA
Distance from cancer center ≤50 km	0.92 (0.90-0.93)	0.94 (0.89-0.99)
Pain score		
High	1.14 (1.10-1.18)	1.01 (0.90-1.13)
Moderate	1.10 (1.07-1.13)	0.96 (0.87-1.05)
Low	1.04 (1.02-1.07)	1.13 (1.04-1.23)
None	1 [Reference]	1 [Reference]
Well-being score		
High	1.15 (1.10-1.19)	1.23 (1.09-1.39)
Moderate	1.09 (1.05-1.13)	1.10 (0.99-1.21)
Low	1.05 (1.02-1.09)	1.01 (0.93-1.10)
None	1 [Reference]	1 [Reference]
Functional score^c^		
10-30	1.31 (1.23-1.39)	1.19 (1.01-1.40)
40-50	1.30 (1.25-1.35)	1.30 (1.15-1.46)
60-70	1.15 (1.11-1.19)	1.41 (1.27-1.56)
80-90	1.08 (1.05-1.11)	1.35 (1.24-1.47)
100	1 [Reference]	1 [Reference]
Appetite		
High, worst appetite	1.36 (1.31-1.41)	1.24 (1.08-1.42)
Moderate	1.22 (1.18-1.26)	1.30 (1.17-1.44)
Low	1.14 (1.11-1.18)	1.16 (1.05-1.27)
No problems, best appetite	1 [Reference]	1 [Reference]
Received home care	1.43 (1.39-1.47)	2.04 (1.87-2.23)
Hospitalized for palliative care	8.00 (7.75-8.26)	17.25 (15.66-19.00)
Resides in long-term care facility	1.58 (1.50-1.67)	1.78 (1.61-1.96)

Abbreviations: CHF, congestive heart failure; COPD, chronic obstructive pulmonary disease; HR, hazard ratio; NA, not applicable.

^a^ A full list of covariates for each model is given in eTable 2 in the Supplement.

^b^ The HR estimates are from the main effects–only model (without the interaction between cancer type and cancer stage).

^c^ Functional score ranges from 0 to 100 (in 10-point increments), with 80 to 100 indicating stable, 40 to 70 indicating transitional, 10 to 30 indicating end of life, and 0 indicating dead.

**Figure 2.**
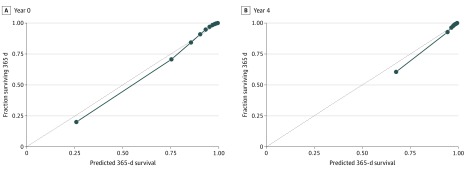
Calibration Plots for Years 0 and 4 Dots represent the deciles of patients’ observed 1-year probability of death plotted against their predicted 1-year probability of death.

Figure 2 gives calibration plots for year 0 and year 4 in the validation cohorts. Model discrimination in the validation cohorts was high. The C index for the 5 yearly models was 0.902 (year 0), 0.912 (year 1), 0.912 (year 2), 0.909 (year 3), and 0.908 (year 4).

To exemplify how the model could be used, we consider the following hypothetical scenario. A 70-year-old man was diagnosed with stage III lung cancer 2 years ago (ie, the calculator would use the year 2 model). His baseline characteristics at year 2 were that he received chemotherapy and radiation therapy in the past (ie, between diagnosis until 3 months ago) and received chemotherapy recently (ie, within the past 3 months) but stopped receiving radiation therapy recently. He had no other chronic conditions, no symptoms except a score of 10 (severe) for worst appetite, and a performance status score of 60 (transitional). For someone with these baseline characteristics in our model, the probability of surviving another 365 days would be 82.4% (95% CI, 80.2%-84.6%) and another 1825 days (5 more years) would be 23.4% (95% CI, 19.2%-28.6%). If the man was hospitalized shortly thereafter, with the use of the same baseline characteristics in the model except indicating a “yes” for a recent hospitalization, the probability of surviving another 365 days would be 71.1% (95% CI, 67.7%-74.5%). If the man experienced adverse effects of chemotherapy and wondered how stopping chemotherapy would affect his long-term survival, with the use of the same baseline characteristics except indicating a “no” for recent chemotherapy, the probability of surviving another 365 days would be 90.1% (95% CI, 88.8%-91.4%). If everything stayed the same and the man lived to 3 years after diagnosis, the probability of surviving another 365 days to year 4 would be 83.8% (95% CI, 81.0%-86.7%) and another 1825 days (to year 8) would be 24.0% (95% CI, 18.3%-31.6%). A first iteration of the PROVIEW calculator is available online.^38^

## Discussion

In this study, we developed and validated a predictive survival model that can be used for all cancer types and incorporates patient-reported outcomes of performance status and symptom severity. By using a large population-based cohort, we achieved high calibration and discrimination. To our knowledge, PROVIEW is the only cancer prognostic model that uses these patient-reported outcomes and updates the risk yearly after diagnosis. Because the covariates are self-reportable by patients and predict risk in days, the model has potential to be a patient-completed online tool, allowing patients to examine survival predictions during various periods as their condition changes.

Compared with other online prognostic tools, such as the UK’s PREDICT for breast cancer^39^ or prostate cancer nomograms,^40^ PROVIEW has features that may make the model easier for patients to use. PROVIEW uses only variables that are easily reported by the patient, whereas other tools require clinical knowledge (eg, biomarkers), which may not be known by patients. For instance, PREDICT uses *ERBB2* (formerly *HER2* or *HER2/neu*) status, estrogen receptor status, and tumor size; the prostate cancer nomograms require knowledge of prostate-specific antigen levels, biopsy cores, or pathology reports. In addition, some tools report mean life expectancy or survival at predetermined periods (eg, 5- and 10-year survival from diagnosis), whereas PROVIEW models survival in days, allowing the user to choose long or short periods in the future. For example, patients at the end of life may be more interested in 30-day survival than 5-year survival. Moreover, because the model was recalculated at each 1-year anniversary and includes symptoms and performance status, it can be used at any time within the first 5 years after diagnosis and accounts for changes in a patient’s condition over time. Some tools have different versions for various posttreatment phases (eg, after radical prostatectomy),^41^ but they do not differentiate among individuals who had the same treatments but have drastically different performance status.

The hypothetical case example described earlier gives potential scenarios in which the model may be useful to inform decision-making and initiate palliative care discussions earlier. In the scenario in which the patient was hospitalized, the 1-year survival risk would decrease. This change in predicted survival may trigger patients and families to review the general outlook of disease trajectory with practitioners, which may lead to discussions about palliative care even though death is not imminent. In the scenario in which the patient considers stopping chemotherapy, the 1-year survival risk would increase. This increase may be associated with the confounding fact that patients who stop chemotherapy might have responded well to treatment, achieved remission, and thus live longer. These nuances need to be discussed with practitioners, along with clinical factors that are not available in the model and preferences and goals of care. Patients can use the model’s survival predictions, which uniquely incorporate changes in symptoms, performance status, treatment, and hospital use along the disease trajectory, to inform discussions and improve decision-making with practitioners.

### Limitations

This study has limitations. Data were not available on genetic biomarkers and specific targeted therapies, which would increase the accuracy of our predictions, particularly for cancer-specific models. Symptom and performance status data at various time points were missing because some patients chose not to voluntarily report them at cancer centers or they did not receive home care assessments and services. Nonetheless, the largest, longitudinal, population-based databases with this information were used. In this version, worsening symptoms and performance status were not considered as outcomes or how they could be modified by other variables. This analysis is planned as a subsequent step, which would further support the model’s usefulness for early palliative care integration. Although the model was validated and the initial online calculator is available, an important next step is to test, validate, and refine the online tool with patient and family users.^42^

## Conclusions

The PROVIEW model appeared to accurately predict changing cancer survival risk over time using administrative clinical data and patient-reported outcomes of symptoms and performance status. Because the model covariates can be completed by patients, PROVIEW may be a useful patient-facing online tool, allowing them to prepare questions around goals of care and treatment preferences before an oncologist visit. In this way, PROVIEW could help patients and families initiate conversations with practitioners about the changing disease trajectory and explore the benefits of palliative care supports earlier.

## Notes

Supplement. **eTable 1.** Baseline Characteristics of Development and Validation Cohorts at Each Year **eTable 2.** Fully Adjusted Main Effects Model Associations for Development Cohort Following Backward Elimination for All Years **eFigure 1.** Calibration Accuracy for 1-Year Survival **eFigure 2.** Kaplan Meier Survival Curve When Patients Grouped by Quintiles of 1-Year Predicted Risk of Death
