# Supplementary material for: Development and Validation of a Prognostic Survival Model With Patient-Reported Outcomes for Patients With Cancer
Source: JAMA Netw Open. 2020 Apr 1;3(4):e201768. doi: 10.1001/jamanetworkopen.2020.1768 (PMC7113728; doi:10.1001/jamanetworkopen.2020.1768)
Supplement: Supplement. — eTable 1. Baseline Characteristics of Development and Validation Cohorts at Each Year eTable 2. Fully Adjusted Main Effects Model Associations for Development Cohort Following Backward Elimination for All Years eFigure 1. Calibration Accuracy for 1-Year Survival eFigure 2. Kaplan Meier Survival Curve When Patients Grouped by Quintiles of 1-Year Predicted Risk of Death [file jamanetwopen-3-e201768-s001.pdf]

Supplementary Online Content

Seow H, Tanuseputro P, Barbera L, et al. Development and validation of a prognostic survival model with patient-reported outcomes for patients with cancer. *JAMA Netw Open*. 2020;3(4):e201768. doi:10.1001/jamanetworkopen.2020.1768

eTable 1. Baseline Characteristics of Development and Validation Cohorts at Each Year

eTable 2. Fully Adjusted Main Effects Model Associations for Development Cohort Following Backward Elimination for All Years

eFigure 1. Calibration Accuracy for 1-Year Survival

eFigure 2. Kaplan Meier Survival Curve When Patients Grouped by Quintiles of 1-Year Predicted Risk of Death

This supplementary material has been provided by the authors to give readers additional information about their work.

© 2020 Seow H et al. *JAMA Network Open.*

eTable 1. Baseline Characteristics of Development and Validation Cohorts at Each Year

		Y0	Y0	Y1	Y1	Y2	Y2	Y3	Y3	Y4	Y4
Variable	Total Cohort	Cohort A (analysis) N=153,296	Cohort B (validation) N=102,198	Cohort A (analysis) N=130,233	Cohort B (validation) N=86,822	Cohort A (analysis) N=110,893	Cohort B (validation) N=73,929	Cohort A (analysis) N=86,189	Cohort B (validation) N=57,460	Cohort A (analysis) N=65,741	Cohort B (validation) N=43,828
**Age at diagnosis**	Mean (SD)	63.66 ± 13.61	63.54 ± 13.62	63.72 ± 13.65	63.73 ± 13.64	64.11 ± 13.58	64.19 ± 13.68	64.71 ± 13.60	64.69 ± 13.45	65.25 ± 13.42	65.27 ± 13.47
	Median (IQR)	65 (55-73)	65 (55-73)	65 (55-73)	65 (55-73)	65 (56-74)	65 (56-74)	66 (56-74)	66 (56-74)	66 (57-75)	67 (57-75)
**Sex**	Female	81,568 (53.2%)	54,131 (53.0%)	71,267 (54.7%)	47,338 (54.5%)	61,656 (55.6%)	40,857 (55.3%)	47,622 (55.3%)	32,055 (55.8%)	36,213 (55.1%)	24,328 (55.5%)
	Male	71,728 (46.8%)	48,067 (47.0%)	58,966 (45.3%)	39,484 (45.5%)	49,237 (44.4%)	33,072 (44.7%)	38,567 (44.7%)	25,405 (44.2%)	29,528 (44.9%)	19,500 (44.5%)
**Cancer type**	Breast	30,855 (20.1%)	20,719 (20.3%)	30,504 (23.4%)	19,901 (22.9%)	27,883 (25.1%)	18,803 (25.4%)	22,327 (25.9%)	15,130 (26.3%)	17,483 (26.6%)	11,618 (26.5%)
	Colorectal	16,776 (10.9%)	11,142 (10.9%)	14,975 (11.5%)	10,071 (11.6%)	12,737 (11.5%)	8,566 (11.6%)	9,871 (11.5%)	6,471 (11.3%)	7,429 (11.3%)	4,928 (11.2%)
	Gynaecological	11,905 (7.8%)	7,879 (7.7%)	10,674 (8.2%)	7,315 (8.4%)	9,412 (8.5%)	6,036 (8.2%)	7,126 (8.3%)	4,837 (8.4%)	5,374 (8.2%)	3,674 (8.4%)
	Head and Neck	6,843 (4.5%)	4,415 (4.3%)	5,888 (4.5%)	3,928 (4.5%)	4,978 (4.5%)	3,320 (4.5%)	3,740 (4.3%)	2,574 (4.5%)	2,872 (4.4%)	1,818 (4.1%)
	Hematology	16,623 (10.8%)	11,052 (10.8%)	14,877 (11.4%)	9,794 (11.3%)	12,747 (11.5%)	8,624 (11.7%)	9,944 (11.5%)	6,597 (11.5%)	7,444 (11.3%)	4,873 (11.1%)
	Lung	19,111 (12.5%)	12,604 (12.3%)	10,720 (8.2%)	7,054 (8.1%)	6,744 (6.1%)	4,468 (6.0%)	4,291 (5.0%)	2,769 (4.8%)	2,802 (4.3%)	1,904 (4.3%)
	Other	14,158 (9.2%)	9,397 (9.2%)	11,868 (9.1%)	8,024 (9.2%)	10,061 (9.1%)	6,707 (9.1%)	7,732 (9.0%)	5,150 (9.0%)	5,701 (8.7%)	3,968 (9.1%)
	Other Gastrointestinal	11,824 (7.7%)	7,879 (7.7%)	6,904 (5.3%)	4,674 (5.4%)	4,609 (4.2%)	2,959 (4.0%)	3,083 (3.6%)	1,998 (3.5%)	2,040 (3.1%)	1,392 (3.2%)
	Other Genitourinary	6,797 (4.4%)	4,611 (4.5%)	5,797 (4.5%)	3,807 (4.4%)	4,720 (4.3%)	3,149 (4.3%)	3,649 (4.2%)	2,429 (4.2%)	2,708 (4.1%)	1,833 (4.2%)
	Prostate	18,404 (12.0%)	12,500 (12.2%)	18,026 (13.8%)	12,254 (14.1%)	17,002 (15.3%)	11,297 (15.3%)	14,426 (16.7%)	9,505 (16.5%)	11,888 (18.1%)	7,820 (17.8%)
**Cancer stage**	Stage 1	32,505 (21.2%)	21,557 (21.1%)	31,646 (24.3%)	21,024 (24.2%)	29,323 (26.4%)	19,597 (26.5%)	24,066 (27.9%)	16,327 (28.4%)	18,845 (28.7%)	12,587 (28.7%)
	Stage 2	34,453 (22.5%)	23,179 (22.7%)	33,180 (25.5%)	22,205 (25.6%)	30,328 (27.3%)	20,314 (27.5%)	24,902 (28.9%)	16,484 (28.7%)	19,704 (30.0%)	13,144 (30.0%)
	Stage 3	24,624 (16.1%)	16,581 (16.2%)	21,264 (16.3%)	14,318 (16.5%)	17,684 (15.9%)	11,776 (15.9%)	13,516 (15.7%)	9,078 (15.8%)	10,162 (15.5%)	6,739 (15.4%)
	Stage 4	22,990 (15.0%)	15,277 (14.9%)	12,633 (9.7%)	8,388 (9.7%)	7,950 (7.2%)	5,181 (7.0%)	5,111 (5.9%)	3,290 (5.7%)	3,415 (5.2%)	2,176 (5.0%)
	Uknown	38,724 (25.3%)	25,604 (25.1%)	31,510 (24.2%)	20,887 (24.1%)	25,608 (23.1%)	17,061 (23.1%)	18,594 (21.6%)	12,281 (21.4%)	13,615 (20.7%)	9,182 (21.0%)
**Radiation in last +3 months**	No	115,715 (75.5%)	77,042 (75.4%)	116,247 (89.3%)	77,741 (89.5%)	106,463 (96.0%)	70,990 (96.0%)	83,397 (96.8%)	55,609 (96.8%)	63,907 (97.2%)	42,669 (97.4%)
	Yes	37,581 (24.5%)	25,156 (24.6%)	13,986 (10.7%)	9,081 (10.5%)	4,430 (4.0%)	2,939 (4.0%)	2,792 (3.2%)	1,851 (3.2%)	1,834 (2.8%)	1,159 (2.6%)
**Chemotherapy in last +3 months**	No	111,810 (72.9%)	74,529 (72.9%)	106,520 (81.8%)	70,975 (81.7%)	96,710 (87.2%)	64,506 (87.3%)	76,971 (89.3%)	51,166 (89.0%)	59,284 (90.2%)	39,537 (90.2%)
	Yes	41,486 (27.1%)	27,669 (27.1%)	23,713 (18.2%)	15,847 (18.3%)	14,183 (12.8%)	9,423 (12.7%)	9,218 (10.7%)	6,294 (11.0%)	6,457 (9.8%)	4,291 (9.8%)
**Cancer surgery in last +3 months**	No	81,817 (53.4%)	54,571 (53.4%)	120,650 (92.6%)	80,523 (92.7%)	105,021 (94.7%)	70,014 (94.7%)	82,232 (95.4%)	54,749 (95.3%)	62,884 (95.7%)	41,921 (95.6%)
	Yes	71,479 (46.6%)	47,627 (46.6%)	9,583 (7.4%)	6,299 (7.3%)	5,872 (5.3%)	3,915 (5.3%)	3,957 (4.6%)	2,711 (4.7%)	2,857 (4.3%)	1,907 (4.4%)
**Past radiation (btwn index and -3mos from present date)**	No	N/A	N/A	72,666 (55.8%)	48,697 (56.1%)	57,301 (51.7%)	38,053 (51.5%)	42,870 (49.7%)	28,639 (49.8%)	31,536 (48.0%)	21,055 (48.0%)
	Yes	N/A	N/A	57,567 (44.2%)	38,125 (43.9%)	53,592 (48.3%)	35,876 (48.5%)	43,319 (50.3%)	28,821 (50.2%)	34,205 (52.0%)	22,773 (52.0%)

© 2020 Seow H et al. *JAMA Network Open.*

**Past chemo (btwn index and -3mos from present date)**	No	N/A	N/A	81,417 (62.5%)	54,304 (62.5%)	67,910 (61.2%)	45,210 (61.2%)	53,200 (61.7%)	35,434 (61.7%)	40,541 (61.7%)	27,111 (61.9%)
	Yes	N/A	N/A	48,816 (37.5%)	32,518 (37.5%)	42,983 (38.8%)	28,719 (38.8%)	32,989 (38.3%)	22,026 (38.3%)	25,200 (38.3%)	16,717 (38.1%)
**Past surgery (btwn index and -3mos from present date)**	No	N/A	N/A	49,301 (37.9%)	32,992 (38.0%)	35,629 (32.1%)	23,669 (32.0%)	25,178 (29.2%)	16,528 (28.8%)	17,769 (27.0%)	11,647 (26.6%)
	Yes	N/A	N/A	80,932 (62.1%)	53,830 (62.0%)	75,264 (67.9%)	50,260 (68.0%)	61,011 (70.8%)	40,932 (71.2%)	47,972 (73.0%)	32,181 (73.4%)
**Distance from regional cancer centre**	<=50 km	121,902 (79.5%)	81,800 (80.0%)	104,438 (80.2%)	69,455 (80.0%)	88,784 (80.1%)	59,469 (80.4%)	69,066 (80.1%)	45,965 (80.0%)	52,582 (80.0%)	34,961 (79.8%)
**Pt hospitalized in the past 3 months?**	Yes	9,682 (6.3%)	6,369 (6.2%)	20,964 (16.1%)	14,022 (16.2%)	12,895 (11.6%)	8,610 (11.6%)	8,809 (10.2%)	5,855 (10.2%)	6,212 (9.4%)	4,166 (9.5%)
**Chronic Diseases**	AMI	546 (0.4%)	309 (0.3%)	580 (0.4%)	363 (0.4%)	306 (0.3%)	253 (0.3%)	223 (0.3%)	167 (0.3%)	193 (0.3%)	133 (0.3%)
	Arrhythmia	11,210 (7.3%)	7,456 (7.3%)	9,263 (7.1%)	6,262 (7.2%)	8,259 (7.4%)	5,541 (7.5%)	6,822 (7.9%)	4,453 (7.7%)	5,344 (8.1%)	3,544 (8.1%)
	Asthma	19,656 (12.8%)	12,885 (12.6%)	17,223 (13.2%)	11,527 (13.3%)	14,966 (13.5%)	9,906 (13.4%)	11,605 (13.5%)	7,807 (13.6%)	8,902 (13.5%)	5,980 (13.6%)
	CHF	8,470 (5.5%)	5,695 (5.6%)	8,549 (6.6%)	5,624 (6.5%)	7,222 (6.5%)	4,959 (6.7%)	5,814 (6.7%)	3,858 (6.7%)	4,566 (6.9%)	2,973 (6.8%)
	COPD	13,567 (8.9%)	9,048 (8.9%)	12,298 (9.4%)	8,135 (9.4%)	9,951 (9.0%)	6,462 (8.7%)	7,580 (8.8%)	5,015 (8.7%)	5,724 (8.7%)	3,830 (8.7%)
	Coronary	22,576 (14.7%)	15,142 (14.8%)	16,328 (12.5%)	10,710 (12.3%)	14,129 (12.7%)	9,240 (12.5%)	11,246 (13.0%)	7,431 (12.9%)	8,697 (13.2%)	5,989 (13.7%)
	Dementia	2,543 (1.7%)	1,613 (1.6%)	2,349 (1.8%)	1,593 (1.8%)	2,262 (2.0%)	1,573 (2.1%)	2,054 (2.4%)	1,384 (2.4%)	1,757 (2.7%)	1,158 (2.6%)
	Diabetes	33,446 (21.8%)	22,630 (22.1%)	29,838 (22.9%)	19,891 (22.9%)	25,886 (23.3%)	17,275 (23.4%)	20,419 (23.7%)	13,732 (23.9%)	15,887 (24.2%)	10,616 (24.2%)
	Hypertension	80,517 (52.5%)	53,525 (52.4%)	71,059 (54.6%)	47,342 (54.5%)	60,963 (55.0%)	40,668 (55.0%)	47,983 (55.7%)	31,988 (55.7%)	37,135 (56.5%)	24,709 (56.4%)
	IBD	970 (0.6%)	660 (0.6%)	834 (0.6%)	512 (0.6%)	734 (0.7%)	471 (0.6%)	597 (0.7%)	386 (0.7%)	460 (0.7%)	307 (0.7%)
	Mental health	5,630 (3.7%)	3,727 (3.6%)	5,281 (4.1%)	3,579 (4.1%)	4,388 (4.0%)	2,890 (3.9%)	3,242 (3.8%)	2,196 (3.8%)	2,392 (3.6%)	1,504 (3.4%)
	Mood Disorder	18,966 (12.4%)	12,593 (12.3%)	21,765 (16.7%)	14,409 (16.6%)	17,778 (16.0%)	11,864 (16.0%)	11,322 (13.1%)	7,434 (12.9%)	7,846 (11.9%)	5,008 (11.4%)
	Osteoarthritis	63,935 (41.7%)	42,318 (41.4%)	45,381 (34.8%)	30,294 (34.9%)	41,133 (37.1%)	27,502 (37.2%)	33,233 (38.6%)	22,339 (38.9%)	26,500 (40.3%)	17,409 (39.7%)
	Osteoporosis	8,173 (5.3%)	5,359 (5.2%)	5,255 (4.0%)	3,483 (4.0%)	4,941 (4.5%)	3,348 (4.5%)	4,267 (5.0%)	2,776 (4.8%)	3,476 (5.3%)	2,330 (5.3%)
	Renal disease	6,866 (4.5%)	4,558 (4.5%)	7,696 (5.9%)	4,980 (5.7%)	6,823 (6.2%)	4,533 (6.1%)	5,447 (6.3%)	3,708 (6.5%)	4,392 (6.7%)	2,854 (6.5%)
	Rheumatoid arthritis	2,846 (1.9%)	1,899 (1.9%)	2,414 (1.9%)	1,717 (2.0%)	2,181 (2.0%)	1,453 (2.0%)	1,697 (2.0%)	1,210 (2.1%)	1,340 (2.0%)	927 (2.1%)
	Stroke	4,243 (2.8%)	2,787 (2.7%)	3,123 (2.4%)	2,135 (2.5%)	2,799 (2.5%)	1,881 (2.5%)	2,334 (2.7%)	1,501 (2.6%)	1,794 (2.7%)	1,249 (2.8%)
**Functional score at index (+3 mo)**	Missing	82,357 (53.7%)	54,649 (53.5%)	76,800 (59.0%)	50,888 (58.6%)	74,565 (67.2%)	49,561 (67.0%)	59,117 (68.6%)	39,333 (68.5%)	46,012 (70.0%)	30,434 (69.4%)
	0	34,314 (22.4%)	23,316 (22.8%)	23,422 (18.0%)	15,685 (18.1%)	18,088 (16.3%)	12,205 (16.5%)	14,248 (16.5%)	9,570 (16.7%)	10,605 (16.1%)	7,222 (16.5%)
	1	21,596 (14.1%)	14,429 (14.1%)	20,281 (15.6%)	13,617 (15.7%)	12,330 (11.1%)	8,217 (11.1%)	8,703 (10.1%)	5,724 (10.0%)	6,099 (9.3%)	4,187 (9.6%)
	2	8,427 (5.5%)	5,487 (5.4%)	5,737 (4.4%)	3,985 (4.6%)	3,441 (3.1%)	2,300 (3.1%)	2,425 (2.8%)	1,703 (3.0%)	1,749 (2.7%)	1,157 (2.6%)
	3	4,893 (3.2%)	3,216 (3.1%)	2,994 (2.3%)	1,973 (2.3%)	1,824 (1.6%)	1,267 (1.7%)	1,272 (1.5%)	855 (1.5%)	977 (1.5%)	627 (1.4%)
	4	1,709 (1.1%)	1,101 (1.1%)	999 (0.8%)	674 (0.8%)	645 (0.6%)	379 (0.5%)	424 (0.5%)	275 (0.5%)	299 (0.5%)	201 (0.5%)
**Pain score at index (+3 mo)**	Missing	62,049 (40.5%)	41,049 (40.2%)	56,641 (43.5%)	37,734 (43.5%)	62,853 (56.7%)	41,599 (56.3%)	52,411 (60.8%)	35,030 (61.0%)	42,978 (65.4%)	28,473 (65.0%)
	0	43,879 (28.6%)	29,311 (28.7%)	38,905 (29.9%)	25,933 (29.9%)	26,630 (24.0%)	18,113 (24.5%)	19,272 (22.4%)	12,666 (22.0%)	12,944 (19.7%)	8,703 (19.9%)
	1	23,550 (15.4%)	16,078 (15.7%)	19,891 (15.3%)	13,264 (15.3%)	12,137 (10.9%)	8,256 (11.2%)	8,213 (9.5%)	5,612 (9.8%)	5,686 (8.6%)	3,800 (8.7%)
	2	15,705 (10.2%)	10,374 (10.2%)	9,827 (7.5%)	6,666 (7.7%)	6,248 (5.6%)	3,967 (5.4%)	4,228 (4.9%)	2,746 (4.8%)	2,753 (4.2%)	1,964 (4.5%)

© 2020 Seow H et al. *JAMA Network Open.*

	3	8,113 (5.3%)	5,386 (5.3%)	4,969 (3.8%)	3,225 (3.7%)	3,025 (2.7%)	1,994 (2.7%)	2,065 (2.4%)	1,406 (2.4%)	1,380 (2.1%)	888 (2.0%)
**Wellbeing score at index (+3 mo)**	Missing	59,535 (38.8%)	39,381 (38.5%)	56,491 (43.4%)	37,644 (43.4%)	62,724 (56.6%)	41,515 (56.2%)	52,329 (60.7%)	34,966 (60.9%)	42,920 (65.3%)	28,398 (64.8%)
	0	23,898 (15.6%)	16,078 (15.7%)	24,948 (19.2%)	16,752 (19.3%)	17,381 (15.7%)	11,663 (15.8%)	12,457 (14.5%)	8,253 (14.4%)	8,417 (12.8%)	5,777 (13.2%)
	1	35,305 (23.0%)	23,905 (23.4%)	28,141 (21.6%)	18,821 (21.7%)	18,337 (16.5%)	12,435 (16.8%)	13,036 (15.1%)	8,618 (15.0%)	8,743 (13.3%)	5,939 (13.6%)
	2	23,941 (15.6%)	15,868 (15.5%)	14,848 (11.4%)	9,796 (11.3%)	8,918 (8.0%)	5,941 (8.0%)	6,128 (7.1%)	4,053 (7.1%)	4,073 (6.2%)	2,711 (6.2%)
	3	10,617 (6.9%)	6,966 (6.8%)	5,805 (4.5%)	3,809 (4.4%)	3,533 (3.2%)	2,375 (3.2%)	2,239 (2.6%)	1,570 (2.7%)	1,588 (2.4%)	1,003 (2.3%)
**Dyspnea score at index (+3 mo)**	Missing	58,577 (38.2%)	38,729 (37.9%)	56,079 (43.1%)	37,392 (43.1%)	62,419 (56.3%)	41,319 (55.9%)	52,063 (60.4%)	34,802 (60.6%)	42,731 (65.0%)	28,284 (64.5%)
	0	85,575 (55.8%)	57,398 (56.2%)	68,882 (52.9%)	45,921 (52.9%)	45,062 (40.6%)	30,375 (41.1%)	31,765 (36.9%)	21,120 (36.8%)	21,426 (32.6%)	14,541 (33.2%)
	1	9,144 (6.0%)	6,071 (5.9%)	5,272 (4.0%)	3,509 (4.0%)	3,412 (3.1%)	2,235 (3.0%)	2,361 (2.7%)	1,538 (2.7%)	1,584 (2.4%)	1,003 (2.3%)
**Depression score at index (+3 mo)**	Missing	62,941 (41.1%)	41,687 (40.8%)	56,993 (43.8%)	37,970 (43.7%)	63,115 (56.9%)	41,772 (56.5%)	52,603 (61.0%)	35,161 (61.2%)	43,128 (65.6%)	28,545 (65.1%)
	0	72,188 (47.1%)	48,355 (47.3%)	61,636 (47.3%)	41,106 (47.3%)	40,515 (36.5%)	27,292 (36.9%)	28,551 (33.1%)	19,043 (33.1%)	19,287 (29.3%)	13,129 (30.0%)
	1	18,167 (11.9%)	12,156 (11.9%)	11,604 (8.9%)	7,746 (8.9%)	7,263 (6.5%)	4,865 (6.6%)	5,035 (5.8%)	3,256 (5.7%)	3,326 (5.1%)	2,154 (4.9%)
**ESAS score at index (+3 mo)**											
**Tiredness**	Missing	70,973 (46.3%)	47,090 (46.1%)	61,073 (46.9%)	40,729 (46.9%)	66,077 (59.6%)	43,736 (59.2%)	54,804 (63.6%)	36,674 (63.8%)	44,692 (68.0%)	29,620 (67.6%)
	Level 1	23,378 (15.3%)	15,511 (15.2%)	19,471 (15.0%)	13,016 (15.0%)	13,927 (12.6%)	9,355 (12.7%)	10,191 (11.8%)	6,599 (11.5%)	6,779 (10.3%)	4,619 (10.5%)
	Level 2	24,604 (16.0%)	17,011 (16.6%)	26,261 (20.2%)	17,398 (20.0%)	16,683 (15.0%)	11,383 (15.4%)	11,726 (13.6%)	7,761 (13.5%)	7,860 (12.0%)	5,368 (12.2%)
	Level 3	20,327 (13.3%)	13,339 (13.1%)	14,758 (11.3%)	10,002 (11.5%)	9,202 (8.3%)	6,051 (8.2%)	6,137 (7.1%)	4,196 (7.3%)	4,142 (6.3%)	2,751 (6.3%)
	Level 4	14,014 (9.1%)	9,247 (9.0%)	8,670 (6.7%)	5,677 (6.5%)	5,004 (4.5%)	3,404 (4.6%)	3,331 (3.9%)	2,230 (3.9%)	2,268 (3.4%)	1,470 (3.4%)
**Drowsiness**	Missing	71,030 (46.3%)	47,139 (46.1%)	61,135 (46.9%)	40,771 (47.0%)	66,104 (59.6%)	43,764 (59.2%)	54,830 (63.6%)	36,685 (63.8%)	44,707 (68.0%)	29,623 (67.6%)
	Level 1	44,081 (28.8%)	29,594 (29.0%)	36,100 (27.7%)	24,173 (27.8%)	24,143 (21.8%)	16,404 (22.2%)	17,213 (20.0%)	11,222 (19.5%)	11,439 (17.4%)	7,699 (17.6%)
	Level 2	19,545 (12.7%)	13,307 (13.0%)	20,012 (15.4%)	13,370 (15.4%)	12,876 (11.6%)	8,545 (11.6%)	8,922 (10.4%)	6,046 (10.5%)	6,076 (9.2%)	4,154 (9.5%)
	Level 3	11,703 (7.6%)	7,572 (7.4%)	8,728 (6.7%)	5,765 (6.6%)	5,316 (4.8%)	3,528 (4.8%)	3,602 (4.2%)	2,416 (4.2%)	2,469 (3.8%)	1,629 (3.7%)
	Level 4	6,937 (4.5%)	4,586 (4.5%)	4,258 (3.3%)	2,743 (3.2%)	2,454 (2.2%)	1,688 (2.3%)	1,622 (1.9%)	1,091 (1.9%)	1,050 (1.6%)	723 (1.6%)
**Nausea**	Missing	70,993 (46.3%)	47,092 (46.1%)	61,106 (46.9%)	40,747 (46.9%)	66,114 (59.6%)	43,758 (59.2%)	54,822 (63.6%)	36,689 (63.9%)	44,703 (68.0%)	29,629 (67.6%)
	Level 1	65,054 (42.4%)	43,679 (42.7%)	56,019 (43.0%)	37,259 (42.9%)	36,926 (33.3%)	24,892 (33.7%)	26,032 (30.2%)	17,226 (30.0%)	17,592 (26.8%)	11,950 (27.3%)
	Level 2	10,698 (7.0%)	7,026 (6.9%)	9,270 (7.1%)	6,198 (7.1%)	5,649 (5.1%)	3,780 (5.1%)	3,910 (4.5%)	2,573 (4.5%)	2,490 (3.8%)	1,622 (3.7%)
	Level 3	4,221 (2.8%)	2,887 (2.8%)	2,721 (2.1%)	1,867 (2.2%)	1,592 (1.4%)	1,078 (1.5%)	1,003 (1.2%)	669 (1.2%)	689 (1.0%)	433 (1.0%)
	Level 4	2,330 (1.5%)	1,514 (1.5%)	1,117 (0.9%)	751 (0.9%)	612 (0.6%)	421 (0.6%)	422 (0.5%)	303 (0.5%)	267 (0.4%)	194 (0.4%)
**Appetite**	Missing	71,032 (46.3%)	47,122 (46.1%)	61,104 (46.9%)	40,749 (46.9%)	66,103 (59.6%)	43,749 (59.2%)	54,824 (63.6%)	36,680 (63.8%)	44,698 (68.0%)	29,623 (67.6%)
	Level 1	40,624 (26.5%)	27,448 (26.9%)	41,400 (31.8%)	27,602 (31.8%)	28,789 (26.0%)	19,499 (26.4%)	21,058 (24.4%)	13,882 (24.2%)	14,525 (22.1%)	9,910 (22.6%)
	Level 2	17,402 (11.4%)	11,691 (11.4%)	15,101 (11.6%)	10,015 (11.5%)	8,981 (8.1%)	6,037 (8.2%)	5,865 (6.8%)	3,909 (6.8%)	3,722 (5.7%)	2,473 (5.6%)
	Level 3	14,364 (9.4%)	9,421 (9.2%)	8,402 (6.5%)	5,671 (6.5%)	4,826 (4.4%)	3,132 (4.2%)	3,039 (3.5%)	2,085 (3.6%)	1,890 (2.9%)	1,261 (2.9%)
	Level 4	9,874 (6.4%)	6,516 (6.4%)	4,226 (3.2%)	2,785 (3.2%)	2,194 (2.0%)	1,512 (2.0%)	1,403 (1.6%)	904 (1.6%)	906 (1.4%)	561 (1.3%)
**Anxiety**	Missing	71,030 (46.3%)	47,105 (46.1%)	61,106 (46.9%)	40,753 (46.9%)	66,106 (59.6%)	43,762 (59.2%)	54,827 (63.6%)	36,681 (63.8%)	44,708 (68.0%)	29,621 (67.6%)
	Level 1	26,933 (17.6%)	18,120 (17.7%)	33,408 (25.7%)	22,238 (25.6%)	22,348 (20.2%)	15,087 (20.4%)	16,025 (18.6%)	10,583 (18.4%)	10,809 (16.4%)	7,325 (16.7%)

© 2020 Seow H et al. *JAMA Network Open.*

	Level 2	26,620 (17.4%)	17,829 (17.4%)	22,308 (17.1%)	14,842 (17.1%)	14,307 (12.9%)	9,637 (13.0%)	9,837 (11.4%)	6,570 (11.4%)	6,559 (10.0%)	4,482 (10.2%)
	Level 3	17,241 (11.2%)	11,540 (11.3%)	9,140 (7.0%)	6,146 (7.1%)	5,578 (5.0%)	3,672 (5.0%)	3,726 (4.3%)	2,469 (4.3%)	2,514 (3.8%)	1,621 (3.7%)
	Level 4	11,472 (7.5%)	7,604 (7.4%)	4,271 (3.3%)	2,843 (3.3%)	2,554 (2.3%)	1,771 (2.4%)	1,774 (2.1%)	1,157 (2.0%)	1,151 (1.8%)	779 (1.8%)
**1ry or 2ry caregiver lives with pt**	Missing	144,069 (94.0%)	96,092 (94.0%)	124,105 (95.3%)	82,652 (95.2%)	107,379 (96.8%)	71,551 (96.8%)	83,892 (97.3%)	55,849 (97.2%)	64,207 (97.7%)	42,756 (97.6%)
	No	2,750 (1.8%)	1,843 (1.8%)	1,907 (1.5%)	1,345 (1.5%)	1,198 (1.1%)	853 (1.2%)	858 (1.0%)	577 (1.0%)	557 (0.8%)	391 (0.9%)
	Yes	6,311 (4.1%)	4,169 (4.1%)	4,092 (3.1%)	2,737 (3.2%)	2,226 (2.0%)	1,485 (2.0%)	1,389 (1.6%)	993 (1.7%)	945 (1.4%)	661 (1.5%)
	NA (i.e. does not have a 1-ry caregiver)	166 (0.1%)	94 (0.1%)	129 (0.1%)	88 (0.1%)	90 (0.1%)	40 (0.1%)	50 (0.1%)	41 (0.1%)	32 (0.0%)	20 (0.0%)
**Pt has regular primary care physician**	No	3,635 (2.4%)	2,319 (2.3%)	1,701 (1.3%)	1,113 (1.3%)	1,698 (1.5%)	1,117 (1.5%)	1,655 (1.9%)	1,080 (1.9%)	1,320 (2.0%)	860 (2.0%)
	Rostered	126,357 (82.4%)	84,473 (82.7%)	109,559 (84.1%)	72,980 (84.1%)	94,190 (84.9%)	62,829 (85.0%)	73,549 (85.3%)	49,169 (85.6%)	56,504 (85.9%)	37,616 (85.8%)
	Virtually rostered	23,304 (15.2%)	15,406 (15.1%)	18,973 (14.6%)	12,729 (14.7%)	15,005 (13.5%)	9,983 (13.5%)	10,985 (12.7%)	7,211 (12.5%)	7,917 (12.0%)	5,352 (12.2%)
**Pt has received physician home visit**	Yes	1,262 (0.8%)	842 (0.8%)	1,110 (0.9%)	748 (0.9%)	641 (0.6%)	417 (0.6%)	373 (0.4%)	248 (0.4%)	252 (0.4%)	165 (0.4%)
**Pt had palliative care hospitalization**	Yes	10,172 (6.6%)	6,581 (6.4%)	7,557 (5.8%)	5,089 (5.9%)	4,280 (3.9%)	2,786 (3.8%)	2,543 (3.0%)	1,640 (2.9%)	1,662 (2.5%)	1,076 (2.5%)
**Pt resides in LTC**	Yes	1,721 (1.1%)	1,096 (1.1%)	1,901 (1.5%)	1,346 (1.6%)	1,584 (1.4%)	1,072 (1.5%)	1,262 (1.5%)	839 (1.5%)	1,040 (1.6%)	690 (1.6%)
**Pt had EoL homecare**	No	144,258 (94.1%)	96,247 (94.2%)	122,800 (94.3%)	81,851 (94.3%)	106,694 (96.2%)	71,324 (96.5%)	83,798 (97.2%)	55,926 (97.3%)	64,234 (97.7%)	42,799 (97.7%)
	Pt has nursing or personal support	9,038 (5.9%)	5,951 (5.8%)	7,433 (5.7%)	4,971 (5.7%)	4,199 (3.8%)	2,605 (3.5%)	2,391 (2.8%)	1,534 (2.7%)	1,507 (2.3%)	1,029 (2.3%)

© 2020 Seow H et al. *JAMA Network Open.*

eTable 2. Fully Adjusted Main Effects Model Associations for Development Cohort Following Backward Elimination for All Years [Outcome=time to death (in days)]

Year from death		Y0				Y1				Y2				Y3				Y4			
**Parameter**	**Level**	**Estimate**	**StdErr**	**Hazard ratio**	**Prob ChiSq**	**Estimate**	**StdErr**	**Hazard ratio**	**Prob ChiSq**	**Estimate**	**StdErr**	**Hazard ratio**	**Prob ChiSq**	**Estimate**	**StdErr**	**Hazard ratio**	**Prob ChiSq**	**Estimate**	**StdErr**	**Hazard ratio**	**Prob ChiSq**
**Age (centered)**	Age at indexdate	0.0267	0.0004	1.03	<.0001	0.0286	4.75E-04	1.03	<.0001	0.0302	0.0006	1.03	<.0001	0.0320	0.0008	1.03	<.0001	0.0335	0.0011	1.03	<.0001
**Age*Age**		0.0001	0.0000	1.00	<.0001	0.0003	2.30E-05	1.00	<.0001	0.0004	0.0000	1.00	<.0001	0.0004	0.0000	1.00	<.0001	0.0004	0.0001	1.00	<.0001
**Sex**	Female	-0.1392	0.0099	0.87	<.0001	-0.1654	0.0130	0.85	<.0001	-0.1620	0.0178	0.85	<.0001	-0.1881	0.0233	0.83	<.0001	-0.1944	0.0308	0.82	<.0001
**Admitted to hospital in last 3 months**	Yes	0.1583	0.0142	1.17	<.0001	0.5015	0.0139	1.65	<.0001	0.5670	0.0190	1.76	<.0001	0.6645	0.0235	1.94	<.0001	0.6640	0.0315	1.94	<.0001
**Chronic diseases**	AMI	N/A	N/A	N/A	N/A	N/A	N/A	N/A	N/A	N/A	N/A	N/A	N/A	N/A	N/A	N/A	N/A	N/A	N/A	N/A	N/A
	Arrythmia	N/A	N/A	N/A	N/A	0.0411	0.0186	1.04	0.0273	N/A	N/A	N/A	N/A	N/A	N/A	N/A	N/A	N/A	N/A	N/A	N/A
	Asthma	-0.0593	0.0129	0.94	<.0001	N/A	N/A	N/A	N/A	-0.0625	0.0210	0.94	0.0029	-0.0752	0.0267	0.93	0.0048	N/A	N/A	N/A	N/A
	CHF	0.1893	0.0153	1.21	<.0001	0.1978	0.0180	1.22	<.0001	0.2643	0.0222	1.30	<.0001	0.2936	0.0274	1.34	<.0001	0.3466	0.0346	1.41	<.0001
	COPD	0.1757	0.0130	1.19	<.0001	0.2019	0.0156	1.22	<.0001	0.2961	0.0208	1.35	<.0001	0.3303	0.0263	1.39	<.0001	0.3209	0.0326	1.38	<.0001
	Coronary	N/A	N/A	N/A	N/A	N/A	N/A	N/A	N/A	N/A	N/A	N/A	N/A	N/A	N/A	N/A	N/A	N/A	N/A	N/A	N/A
	Dementia	0.1526	0.0258	1.17	<.0001	0.2781	0.0297	1.32	<.0001	0.3250	0.0351	1.38	<.0001	0.3025	0.0400	1.35	<.0001	0.4891	0.0483	1.63	<.0001
	Diabetes	0.0435	0.0098	1.04	<.0001	0.0451	0.0123	1.05	0.0002	0.0474	0.0160	1.05	0.0030	0.0818	0.0204	1.09	<.0001	N/A	N/A	N/A	N/A
	Hypertension	N/A	N/A	N/A	N/A	N/A	N/A	N/A	N/A	N/A	N/A	N/A	N/A	N/A	N/A	N/A	N/A	N/A	N/A	N/A	N/A
	IBD	N/A	N/A	N/A	N/A	0.1798	0.0630	1.20	0.0043	N/A	N/A	N/A	N/A	N/A	N/A	N/A	N/A	N/A	N/A	N/A	N/A
	Mental health	N/A	N/A	N/A	N/A	N/A	N/A	N/A	N/A	N/A	N/A	N/A	N/A	0.09105	0.04242	1.10	0.0318	N/A	N/A	N/A	N/A
	Mood Disorder	-0.0392	0.0131	0.96	0.0027	N/A	N/A	N/A	N/A	N/A	N/A	N/A	N/A	N/A	N/A	N/A	N/A	N/A	N/A	N/A	N/A
	Osteoarthritis	-0.0727	0.0088	0.93	<.0001	-0.0849	0.0115	0.92	<.0001	-0.1103	0.0149	0.90	<.0001	-0.1252	0.0191	0.88	<.0001	-0.07161	0.02446	0.93	0.0034
	Osteoporosis	-0.0536	0.0185	0.95	0.0038	-0.0860	0.0276	0.92	0.0018	-0.0754	0.0347	0.93	0.0300	-0.1243	0.0421	0.88	0.0032	N/A	N/A	N/A	N/A
	Renal disease	0.1382	0.0172	1.15	<.0001	0.1595	0.0186	1.17	<.0001	0.2442	0.0230	1.28	<.0001	0.2317	0.0288	1.26	<.0001	0.23207	0.03544	1.26	<.0001
	Rheumatoid arthritis	0.0837	0.0278	1.09	0.0026	N/A	N/A	N/A	N/A	N/A	N/A	N/A	N/A	0.16964	0.05748	1.19	0.0032	N/A	N/A	N/A	N/A
	Stroke	0.1167	0.0212	1.12	<.0001	0.0727	0.0285	1.08	0.0108	0.1258	0.0352	1.13	0.0003	0.1491	0.0429	1.16	0.0005	N/A	N/A	N/A	N/A
**Cancer type (ref=lung)***	Prostate	-2.0793	0.0888	.	<.0001	-1.9389	0.0967	.	<.0001	-1.7970	0.1125	.	<.0001	-1.5953	0.1367	.	<.0001	-1.5414	0.1770	.	<.0001
	Other Genitourinary	-0.7660	0.0743	.	<.0001	-0.7263	0.0798	.	<.0001	-0.7355	0.0952	.	<.0001	-0.6226	0.1181	.	<.0001	-0.7979	0.1396	.	<.0001
	Other Gastrointestinal	0.0079	0.0570	.	0.8905	-0.0534	0.0675	.	0.4291	-0.0542	0.0857	.	0.5268	0.0568	0.1069	.	0.5952	0.0198	0.1356	.	0.8840
	Other	-1.0549	0.0598	.	<.0001	-0.8944	0.0647	.	<.0001	-0.7753	0.0771	.	<.0001	-0.6180	0.0938	.	<.0001	-0.5226	0.1169	.	<.0001
	Hematology	-1.0768	0.0923	.	<.0001	-1.0423	0.1003	.	<.0001	-0.8451	0.1133	.	<.0001	-0.7160	0.1364	.	<.0001	-0.8673	0.1786	.	<.0001
	Head and Neck	-0.9271	0.0731	.	<.0001	-0.8124	0.0814	.	<.0001	-0.6582	0.0959	.	<.0001	-0.5241	0.1133	.	<.0001	-0.5561	0.1424	.	<.0001
	Gynaecological	-1.0148	0.0481	.	<.0001	-0.8141	0.0522	.	<.0001	-0.6837	0.0610	.	<.0001	-0.6444	0.0785	.	<.0001	-0.6521	0.0987	.	<.0001

© 2020 Seow H et al. *JAMA Network Open.*

	Colorectal	-0.7025	0.0519	.	<.0001	-0.6671	0.0575	.	<.0001	-0.5464	0.0673	.	<.0001	-0.3803	0.0807	.	<.0001	-0.4595	0.1001	.	<.0001
	Breast	-1.8515	0.0471	.	<.0001	-1.6542	0.0502	.	<.0001	-1.5456	0.0597	.	<.0001	-1.3351	0.0721	.	<.0001	-1.1950	0.0879	.	<.0001
**Cancer stage (ref=stage 1)***	Unk	0.7022	0.0407	.	<.0001	0.3113	0.0524	.	<.0001	0.2442	0.0712	.	0.0006	0.0168	0.0942	.	0.8586	0.1756	0.1260	.	0.1633
	4	1.1931	0.0294	.	<.0001	0.6277	0.0377	.	<.0001	0.2925	0.0539	.	<.0001	0.3471	0.0805	.	<.0001	-0.0384	0.1149	.	0.7385
	3	0.8134	0.0312	.	<.0001	0.4474	0.0382	.	<.0001	0.2173	0.0512	.	<.0001	0.2332	0.0715	.	0.0011	-0.1633	0.1017	.	0.1081
	2	0.4030	0.0417	.	<.0001	0.1801	0.0498	.	0.0003	-0.0224	0.0661	.	0.7346	0.0113	0.0890	.	0.8986	0.0558	0.1190	.	0.6389
**Cancer stage : cancer type (ref=lung : stage 1)***																					
cancer_stage Unk * Prostate		0.8261	0.1123	.	<.0001	1.0522	0.1245	.	<.0001	1.0075	0.1486	.	<.0001	1.2284	0.1832	.	<.0001	0.6627	0.2459	.	0.0070
cancer_stage Unk * Other Genitourinary		0.3318	0.0844	.	<.0001	0.4620	0.0954	.	<.0001	0.3772	0.1193	.	0.0016	0.5636	0.1511	.	0.0002	0.4888	0.1888	.	0.0096
cancer_stage Unk * Other Gastrointestinal		0.0662	0.0670	.	0.3233	0.0731	0.0840	.	0.3842	-0.0376	0.1122	.	0.7376	0.0776	0.1461	.	0.5956	-0.1414	0.1943	.	0.4667
cancer_stage Unk * Other		0.7636	0.0693	.	<.0001	0.7242	0.0804	.	<.0001	0.4933	0.1026	.	<.0001	0.5941	0.1299	.	<.0001	0.1990	0.1687	.	0.2381
cancer_stage Unk * Hematology		0.1194	0.0984	.	0.225	0.3308	0.1103	.	0.0027	0.2169	0.1302	.	0.0956	0.4232	0.1606	.	0.0084	0.3730	0.2123	.	0.0789
cancer_stage Unk * Head and Neck		0.2159	0.0860	.	0.012	0.3774	0.1017	.	0.0002	0.2346	0.1275	.	0.0656	0.4129	0.1585	.	0.0092	0.3781	0.2062	.	0.0668
cancer_stage Unk*Gynaecological		0.4857	0.0658	.	<.0001	0.6265	0.0784	.	<.0001	0.4544	0.1020	.	<.0001	0.6002	0.1340	.	<.0001	0.3969	0.1717	.	0.0208
cancer_stage Unk * Colorectal		0.0795	0.0764	.	0.2982	0.3465	0.0905	.	0.0001	0.2356	0.1161	.	0.0425	0.1785	0.1511	.	0.2377	0.2356	0.1912	.	0.2179
cancer_stage Unk * Breast		0.8072	0.0753	.	<.0001	0.8959	0.0867	.	<.0001	0.8956	0.1074	.	<.0001	0.9724	0.1336	.	<.0001	0.6916	0.1711	.	<.0001
cancer_stage 4 * Prostate		1.0887	0.0951	.	<.0001	1.3720	0.1049	.	<.0001	1.4075	0.1267	.	<.0001	1.1665	0.1620	.	<.0001	1.4617	0.2165	.	<.0001
cancer_stage 4 * Other Genitourinary		0.6675	0.0830	.	<.0001	0.6934	0.0973	.	<.0001	0.8658	0.1278	.	<.0001	0.6434	0.1686	.	0.0001	0.9033	0.2259	.	<.0001
cancer_stage 4 * Other Gastrointestinal		0.0950	0.0613	.	0.1213	0.1055	0.0797	.	0.1857	0.0329	0.1153	.	0.7757	-0.2047	0.1594	.	0.1990	0.1302	0.2240	.	0.5610
cancer_stage 4 * Other		0.6712	0.0751	.	<.0001	0.7634	0.0930	.	<.0001	0.6368	0.1303	.	<.0001	0.3868	0.1833	.	0.0348	0.5207	0.2660	.	0.0503
cancer_stage 4 * Hematology		-0.3104	0.1041	.	0.0029	-0.0913	0.1186	.	0.4414	-0.0585	0.1445	.	0.6859	-0.0679	0.1775	.	0.7021	0.4674	0.2307	.	0.0428
cancer_stage 4 * Head and Neck		-0.1505	0.0816	.	0.0652	0.2068	0.0951	.	0.0297	0.2871	0.1191	.	0.0159	-0.0613	0.1534	.	0.6896	0.5096	0.1982	.	0.0101
cancer_stage 4 * Gynaecological		0.7880	0.0623	.	<.0001	0.9885	0.0764	.	<.0001	0.6694	0.1053	.	<.0001	0.7348	0.1576	.	<.0001	1.0518	0.2263	.	<.0001
cancer_stage 4 * Colorectal		0.5380	0.0566	.	<.0001	0.6991	0.0657	.	<.0001	0.8093	0.0843	.	<.0001	0.6022	0.1143	.	<.0001	0.9047	0.1594	.	<.0001
cancer_stage 4 * Breast		1.2945	0.0597	.	<.0001	1.4229	0.0677	.	<.0001	1.4730	0.0863	.	<.0001	1.1878	0.1159	.	<.0001	1.1296	0.1580	.	<.0001
cancer_stage 3 * Prostate		-0.3055	0.1096	.	0.0053	0.1914	0.1168	.	0.1013	0.4066	0.1369	.	0.0030	0.4518	0.1651	.	0.0062	0.9395	0.2125	.	<.0001
cancer_stage 3 * Other Genitourinary		0.3100	0.0976	.	0.0015	0.5083	0.1078	.	<.0001	0.6123	0.1364	.	<.0001	0.4749	0.1766	.	0.0072	0.8612	0.2304	.	0.0002
cancer_stage 3 * Other Gastrointestinal		0.0876	0.0665	.	0.1877	0.1409	0.0828	.	0.0890	0.2340	0.1141	.	0.0402	-0.0935	0.1612	.	0.5621	0.3244	0.2136	.	0.1289
cancer_stage 3 * Other		0.3740	0.0786	.	<.0001	0.4995	0.0880	.	<.0001	0.5468	0.1093	.	<.0001	0.3743	0.1410	.	0.0079	0.5281	0.1903	.	0.0055
cancer_stage 3 * Hematology		-0.1611	0.1135	.	0.1559	0.0268	0.1254	.	0.8309	0.0114	0.1501	.	0.9395	0.0235	0.1904	.	0.9016	0.4671	0.2414	.	0.0530
cancer_stage 3 * Head and Neck		-0.1653	0.0954	.	0.0832	0.1691	0.1109	.	0.1274	0.1835	0.1376	.	0.1824	0.1624	0.1722	.	0.3456	0.5350	0.2195	.	0.0148

© 2020 Seow H et al. *JAMA Network Open.*

cancer_stage 3 * Gynaecological		0.7298	0.0578	.	<.0001	0.8016	0.0653	.	<.0001	0.8317	0.0810	.	<.0001	0.7638	0.1086	.	<.0001	0.7485	0.1511	.	<.0001
cancer_stage 3 * Colorectal		-0.4427	0.0591	.	<.0001	-0.1824	0.0668	.	0.0063	-0.0315	0.0816	.	0.6994	-0.1459	0.1036	.	0.1594	0.2072	0.1377	.	0.1323
cancer_stage 3 * Breast		0.7947	0.0578	.	<.0001	0.9299	0.0628	.	<.0001	1.2190	0.0768	.	<.0001	0.9676	0.0989	.	<.0001	1.1345	0.1314	.	<.0001
cancer_stage 2 * Prostate		0.0945	0.0983	.	0.3362	0.3693	0.1078	.	0.0006	0.5805	0.1285	.	<.0001	0.5279	0.1593	.	0.0009	0.5131	0.2075	.	0.0134
cancer_stage 2 * Other Genitourinary		0.6397	0.0979	.	<.0001	0.5083	0.1100	.	<.0001	0.7398	0.1391	.	<.0001	0.6317	0.1754	.	0.0003	0.5816	0.2281	.	0.0108
cancer_stage 2 * Other Gastrointestinal		0.1916	0.0731	.	0.0087	0.3607	0.0871	.	<.0001	0.3849	0.1155	.	0.0009	0.1859	0.1524	.	0.2225	-0.0920	0.2016	.	0.6484
cancer_stage 2 * Other		0.3464	0.0822	.	<.0001	0.4243	0.0916	.	<.0001	0.5498	0.1124	.	<.0001	0.4939	0.1421	.	0.0005	0.0410	0.1901	.	0.8294
cancer_stage 2 * Hematology		-0.2168	0.1251	.	0.0831	-0.1698	0.1397	.	0.2243	-0.1956	0.1722	.	0.2560	-0.1328	0.2073	.	0.5218	-0.3358	0.2813	.	0.2327
cancer_stage 2 * Head and Neck		-0.0218	0.1027	.	0.8318	0.1975	0.1165	.	0.0901	0.3601	0.1395	.	0.0098	0.0930	0.1721	.	0.5891	0.1859	0.2201	.	0.3982
cancer_stage 2 * Gynaecological		0.1929	0.0806	.	0.0168	0.3192	0.0888	.	0.0003	0.3940	0.1104	.	0.0004	0.4099	0.1415	.	0.0038	0.2346	0.1838	.	0.2019
cancer_stage 2 * Colorectal		-0.3544	0.0691	.	<.0001	-0.1018	0.0775	.	0.1892	0.0171	0.0958	.	0.8581	-0.1065	0.1203	.	0.3763	-0.1015	0.1550	.	0.5125
cancer_stage 2 * Breast		0.3814	0.0632	.	<.0001	0.4508	0.0694	.	<.0001	0.7290	0.0865	.	<.0001	0.6395	0.1100	.	<.0001	0.3241	0.1418	.	0.0223
**Radiation**	Yes	0.1105	0.0103	1.12	<.0001	0.2821	0.0159	1.33	<.0001	0.4955	0.0243	1.64	<.0001	0.5025	0.0317	1.65	<.0001	0.3885	0.0421	1.48	<.0001
**Chemotherapy**	Yes	0.0617	0.0101	1.06	<.0001	0.3912	0.0148	1.48	<.0001	0.6156	0.0206	1.85	<.0001	0.7828	0.0266	2.19	<.0001	0.7769	0.0351	2.18	<.0001
**Surgery**	Yes	-0.2672	0.0109	0.77	<.0001	-0.1238	0.0211	0.88	<.0001	-0.0737	0.0300	0.93	0.0140	N/A	N/A	N/A	N/A	N/A	N/A	N/A	N/A
**Previous Radiation**	Yes	N/A	N/A	N/A	N/A	N/A	N/A	N/A	N/A	0.0439	0.0160	1.05	0.0060	0.0539	0.0204	1.06	0.0081	0.0766	0.0266	1.08	0.0039
**Previous Chemotherapy**	Yes	N/A	N/A	N/A	N/A	0.1246	0.0136	1.13	<.0001	0.0871	0.0179	1.09	<.0001	0.0711	0.0228	1.07	0.0018	0.1212	0.0295	1.13	<.0001
**Previous Surgery**	Yes	N/A	N/A	N/A	N/A	-0.2129	0.0135	0.81	<.0001	-0.1051	0.0180	0.90	<.0001	-0.0852	0.0237	0.92	0.0003	-0.0759	0.0309	0.93	0.0141
**Distance from cancer centre**	Within 50 km	-0.0891	0.0101	0.92	<.0001	-0.0823	0.0131	0.92	<.0001	-0.0845	0.0170	0.92	<.0001	-0.0580	0.0222	0.94	0.0090	-0.0630	0.0287	0.94	0.0282
**Pain score (ref=Level 0)**	NA	0.0163	0.0414	1.02	0.6944	0.0589	0.0791	1.06	0.4569	-0.0890	0.0981	0.92	0.3642	-0.3053	0.0822	0.74	0.0002	-0.3887	0.1005	0.68	0.0001
	3	0.1322	0.0178	1.14	<.0001	0.1571	0.0253	1.17	<.0001	-0.0156	0.0344	0.99	0.6507	0.0472	0.0448	1.05	0.2919	0.0085	0.0590	1.01	0.8857
	2	0.0964	0.0146	1.10	<.0001	0.1057	0.0203	1.11	<.0001	0.0675	0.0274	1.07	0.0137	0.0989	0.0356	1.10	0.0055	-0.0418	0.0471	0.96	0.3746
	1	0.0422	0.0135	1.04	0.0018	0.0523	0.0174	1.05	0.0026	0.0785	0.0238	1.08	0.0010	0.0415	0.0313	1.04	0.1843	0.1231	0.0410	1.13	0.0027
**Wellbeing score (ref=Level 0)**	NA	0.1816	0.0472	1.20	0.0001	0.1736	0.0674	1.19	0.0100	0.2031	0.0785	1.23	0.0097	0.1847	0.0837	1.20	0.0274	0.1287	0.0994	1.14	0.1956
	3	0.1363	0.0206	1.15	<.0001	0.0538	0.0285	1.06	0.0586	0.1970	0.0373	1.22	<.0001	0.2102	0.0515	1.23	<.0001	0.2057	0.0628	1.23	0.0011
	2	0.0865	0.0169	1.09	<.0001	0.0463	0.0222	1.05	0.0371	0.0983	0.0287	1.10	0.0006	0.1625	0.0397	1.18	<.0001	0.0913	0.0498	1.10	0.0669
	1	0.0520	0.0157	1.05	0.0009	0.0151	0.0190	1.02	0.4272	0.0305	0.0248	1.03	0.2189	0.0708	0.0337	1.07	0.0357	0.0123	0.0441	1.01	0.7799
**Dyspnea score (ref=Level 0)**	NA	-0.3657	0.0589	0.69	<.0001	-0.3932	0.0986	0.68	<.0001	-0.2952	0.1164	0.74	0.0113	N/A	N/A	N/A	N/A	N/A	N/A	N/A	N/A
	1	0.0338	0.0149	1.03	0.023	0.0358	0.0213	1.04	0.0921	0.0946	0.0281	1.10	0.0008	N/A	N/A	N/A	N/A	N/A	N/A	N/A	N/A
**Depression score (ref=Level 0)**	NA	N/A	N/A	N/A	N/A	N/A	N/A	N/A	N/A	N/A	N/A	N/A	N/A	N/A	N/A	N/A	N/A	N/A	N/A	N/A	N/A
	1	N/A	N/A	N/A	N/A	N/A	N/A	N/A	N/A	N/A	N/A	N/A	N/A	N/A	N/A	N/A	N/A	N/A	N/A	N/A	N/A
	NA	0.0336	0.0142	1.03	0.0178	0.0534	0.0193	1.06	0.0056	0.0148	0.0259	1.02	0.5685	-0.0218	0.0336	0.98	0.5162	0.0170	0.0501	1.02	0.7350

© 2020 Seow H et al. *JAMA Network Open.*

**Funtional score (ref=Level 0)**	4	0.2690	0.0309	1.31	<.0001	0.2813	0.0408	1.33	<.0001	0.2825	0.0525	1.33	<.0001	-0.0023	0.0677	1.00	0.9730	0.1727	0.0839	1.19	0.0395
	3	0.2629	0.0208	1.30	<.0001	0.2047	0.0293	1.23	<.0001	0.2032	0.0390	1.23	<.0001	0.2113	0.0481	1.24	<.0001	0.2613	0.0606	1.30	<.0001
	2	0.1381	0.0176	1.15	<.0001	0.1331	0.0244	1.14	<.0001	0.1743	0.0326	1.19	<.0001	0.1171	0.0416	1.12	0.0049	0.3443	0.0518	1.41	<.0001
	1	0.0744	0.0146	1.08	<.0001	0.0873	0.0198	1.09	<.0001	0.1601	0.0262	1.17	<.0001	0.1700	0.0328	1.19	<.0001	0.3025	0.0427	1.35	<.0001
**anxiety (ref=Level 0)**	NA	0.0057	0.1196	1.01	0.9619	N/A	N/A	N/A	N/A	N/A	N/A	N/A	N/A	N/A	N/A	N/A	N/A	0.0407	0.1394	1.04	0.7703
	3	-0.0961	0.0179	0.91	<.0001	N/A	N/A	N/A	N/A	N/A	N/A	N/A	N/A	N/A	N/A	N/A	N/A	0.1162	0.0746	1.12	0.1194
	2	-0.0808	0.0155	0.92	<.0001	N/A	N/A	N/A	N/A	N/A	N/A	N/A	N/A	N/A	N/A	N/A	N/A	-0.0277	0.0543	0.97	0.6101
	1	-0.0601	0.0142	0.94	<.0001	N/A	N/A	N/A	N/A	N/A	N/A	N/A	N/A	N/A	N/A	N/A	N/A	0.1084	0.0437	1.11	0.0131
**appetite (ref=Level 0)**	NA	-0.0547	0.1125	0.95	0.6268	0.2035	0.0509	1.23	<.0001	0.3962	0.0739	1.49	<.0001	0.5324	0.1010	1.70	<.0001	0.4499	0.1169	1.57	0.0001
	3	0.3068	0.0181	1.36	<.0001	0.2263	0.0293	1.25	<.0001	0.1885	0.0420	1.21	<.0001	0.2196	0.0554	1.25	<.0001	0.2117	0.0706	1.24	0.0027
	2	0.1982	0.0160	1.22	<.0001	0.2058	0.0224	1.23	<.0001	0.2440	0.0309	1.28	<.0001	0.0803	0.0416	1.08	0.0534	0.2607	0.0538	1.30	<.0001
	1	0.1309	0.0155	1.14	<.0001	0.1060	0.0191	1.11	<.0001	0.1588	0.0263	1.17	<.0001	0.1652	0.0346	1.18	<.0001	0.1449	0.0468	1.16	0.0019
**nausea (ref=Level 0)**	NA	N/A	N/A	N/A	N/A	-0.0046	0.0623	1.00	0.9406	-0.1378	0.0762	0.87	0.0704	-0.4121	0.1378	0.66	0.0028	-0.0192	0.1519	0.98	0.8992
	3	N/A	N/A	N/A	N/A	0.1167	0.0464	1.12	0.0118	0.0782	0.0664	1.08	0.2392	0.1252	0.0907	1.13	0.1673	0.0744	0.1143	1.08	0.5152
	2	N/A	N/A	N/A	N/A	0.0579	0.0302	1.06	0.0555	0.0649	0.0425	1.07	0.1267	0.0837	0.0587	1.09	0.1534	0.3587	0.0749	1.43	<.0001
	1	N/A	N/A	N/A	N/A	0.0585	0.0196	1.06	0.0029	0.0583	0.0273	1.06	0.0326	0.0358	0.0363	1.04	0.3248	0.0795	0.0484	1.08	0.1010
**drowsiness (ref=Level 0)**	NA	-0.0427	0.1181	0.96	0.7179	N/A	N/A	N/A	N/A	N/A	N/A	N/A	N/A	-0.2927	0.1412	0.75	0.0382	N/A	N/A	N/A	N/A
	3	0.1688	0.0215	1.18	<.0001	N/A	N/A	N/A	N/A	N/A	N/A	N/A	N/A	-0.0867	0.0631	0.92	0.1692	N/A	N/A	N/A	N/A
	2	0.0692	0.0174	1.07	<.0001	N/A	N/A	N/A	N/A	N/A	N/A	N/A	N/A	-0.1068	0.0458	0.90	0.0197	N/A	N/A	N/A	N/A
	1	0.0106	0.0149	1.01	0.4804	N/A	N/A	N/A	N/A	N/A	N/A	N/A	N/A	0.0110	0.0356	1.01	0.7570	N/A	N/A	N/A	N/A
**tiredness (ref=Level 0)**	NA	0.2501	0.1364	1.28	0.0668	0.0509	0.0522	1.05	0.3290	N/A	N/A	N/A	N/A	0.4501	0.1103	1.57	<.0001	N/A	N/A	N/A	N/A
	3	0.2004	0.0228	1.22	<.0001	0.0767	0.0289	1.08	0.0079	N/A	N/A	N/A	N/A	0.2045	0.0601	1.23	0.0007	N/A	N/A	N/A	N/A
	2	0.1602	0.0194	1.17	<.0001	0.0352	0.0247	1.04	0.1547	N/A	N/A	N/A	N/A	0.1496	0.0484	1.16	0.0020	N/A	N/A	N/A	N/A
	1	0.0881	0.0179	1.09	<.0001	0.0690	0.0217	1.07	0.0014	N/A	N/A	N/A	N/A	0.0944	0.0420	1.10	0.0244	N/A	N/A	N/A	N/A
**Homecare (ref=pt has caregiver, does not live w/ pt)**	Missing	-0.0527	0.0249	0.95	0.0345	-0.0529	0.0321	0.95	0.0999	0.0018	0.0435	1.00	0.9676	-0.0684	0.0536	0.93	0.2018	N/A	N/A	N/A	N/A
	NA (pt does not have caregiver)	0.0096	0.1048	1.01	0.9269	0.0841	0.1223	1.09	0.4918	0.2298	0.1566	1.26	0.1422	0.6050	0.2061	1.83	0.0033	N/A	N/A	N/A	N/A
	Yes (primary or secondary caregiver lives with pt)	0.0890	0.0276	1.09	0.0012	0.0752	0.0343	1.08	0.0284	0.1105	0.0462	1.12	0.0167	0.1087	0.0579	1.12	0.0605	N/A	N/A	N/A	N/A
**EoL homecare (ref=pt does not have homecare)**	Pt has homecare: nursing and/or personal support	0.3554	0.0147	1.43	<.0001	0.5074	0.0186	1.66	<.0001	0.5377	0.0258	1.71	<.0001	0.5334	0.0351	1.71	<.0001	0.7147	0.0448	2.04	<.0001
**Primary care physician**	Pt is not rostered	-0.1991	0.0246	0.82	<.0001	-0.0966	0.0451	0.91	0.0322	-0.1562	0.0550	0.86	0.0045	N/A	N/A	N/A	N/A	N/A	N/A	N/A	N/A

© 2020 Seow H et al. *JAMA Network Open.*

**Palliative care (acute)**	Patient had palliative care in acute care setting	2.0790	0.0163	8.00	<.0001	2.5748	0.0221	13.13	<.0001	2.7134	0.0300	15.08	<.0001	2.8141	0.0393	16.68	<.0001	2.8479	0.0493	17.25	<.0001
**Palliative care (home)**	Patient had a home visit by a family physician	N/A	N/A	N/A	N/A	0.2274	0.0335	1.26	<.0001	0.1201	0.0456	1.13	0.0085	N/A	N/A	N/A	N/A	N/A	N/A	N/A	N/A
**LTC**	Patient is a resident of long term care	0.4577	0.0281	1.58	<.0001	0.4998	0.0284	1.65	<.0001	0.5687	0.0349	1.77	<.0001	0.6528	0.0418	1.92	<.0001	0.5756	0.0503	1.78	<.0001

* Hazard ratios are not shown because of the 2-way interaction between cancer type and cancer stage, meaning the association between cancer types and mortality varies for each level of stage, and vice versa; note the hazards are compared to the reference group (lung and stage 1 respectively).

© 2020 Seow H et al. *JAMA Network Open.*

eFigure 1. Calibration Accuracy for 1-Year Survival

eFigure 1a. Year 0 Calibration accuracy for the 1-year survival probability. Patients grouped by deciles of predicted risk. C-index=0.902

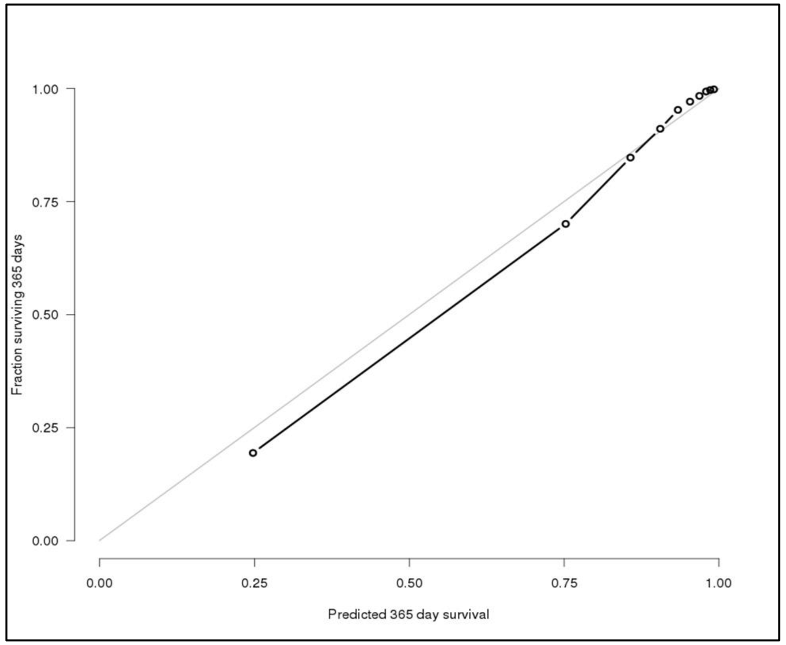

eFigure 1b. Year 1 Calibration accuracy for the 1-year survival probability. Patients grouped by deciles of predicted risk. C-index=0.912

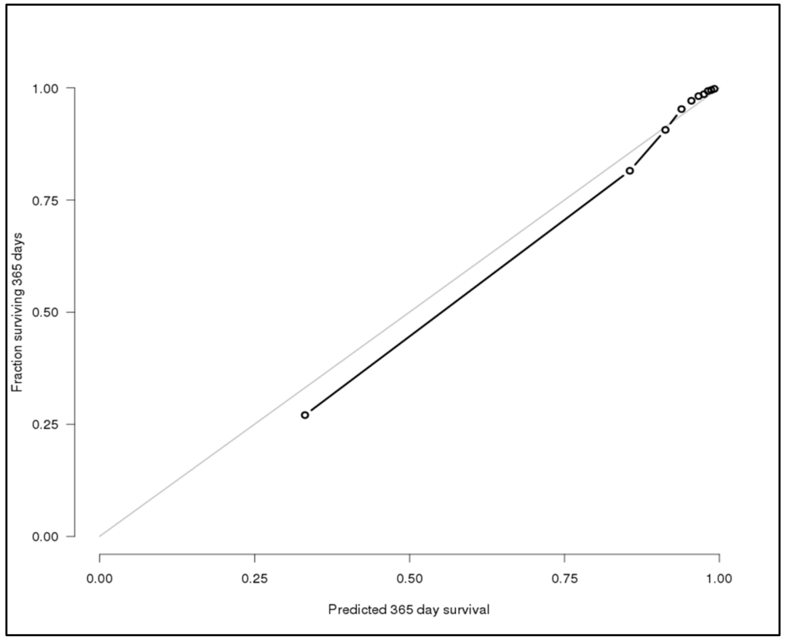

eFigure 1c. Year 2 Calibration accuracy for the 1-year survival probability. Patients grouped by deciles of predicted risk. C-index=0.912

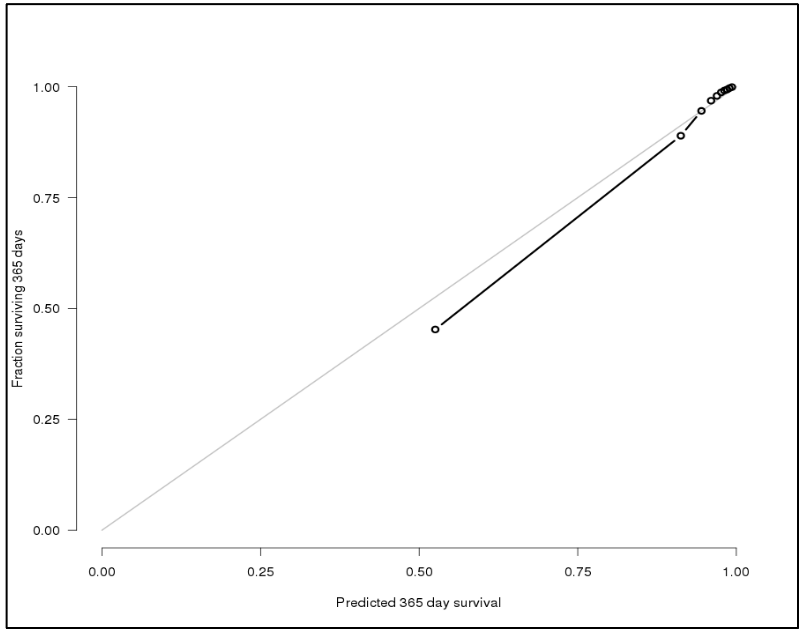

eFigure 1d. Year 3 Calibration accuracy for the 1-year survival probability. Patients grouped by deciles of predicted risk. C-index=0.909

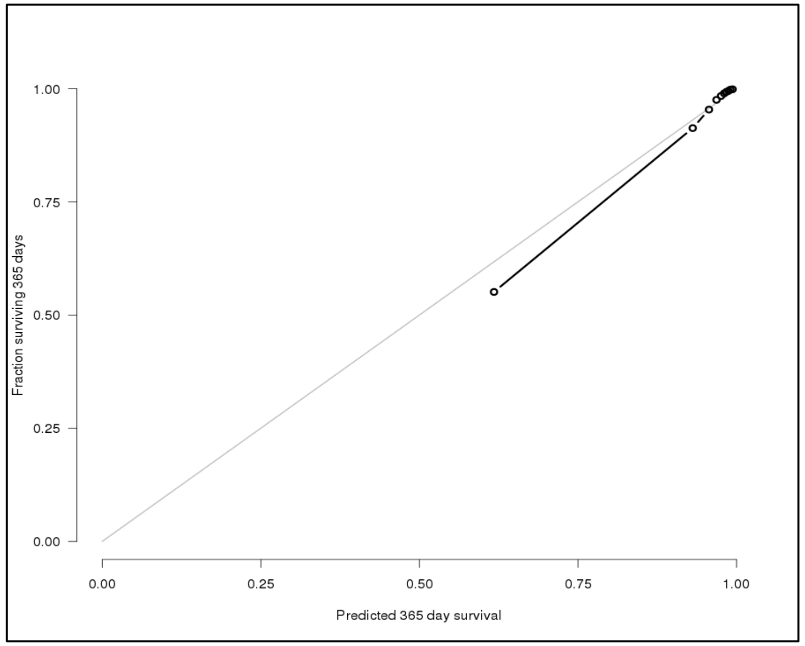

eFigure 1e. Year 4 Calibration accuracy for the 1-year survival probability. Patients grouped by deciles of predicted risk. C-index=0.908

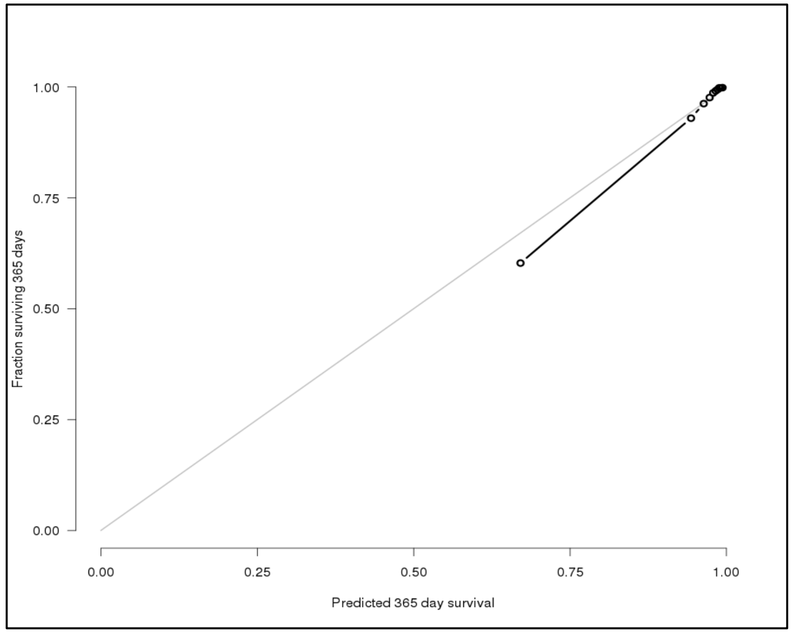

eFigure 1f. Year 0 Calibration accuracy for the 5-year survival probability. Patients group by deciles of predicted risk. C-index=0.835

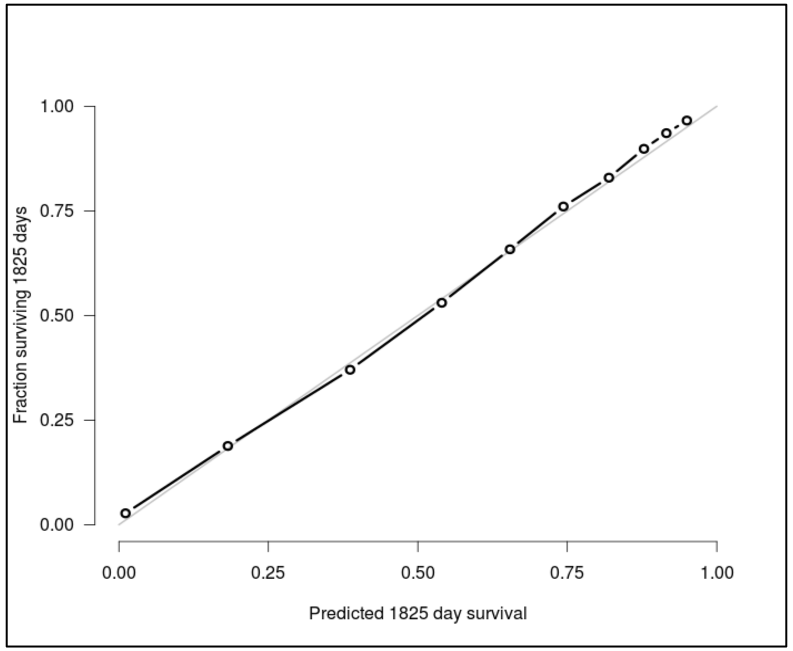

© 2020 Seow H et al. *JAMA Network Open.*

eFigure 2. Kaplan Meier Survival Curve When Patients Grouped by Quintiles of 1-Year Predicted Risk of Death

eFigure 2a. Year 0 – Kaplan Meier survival curve when patients grouped by quintiles of 1-year predicted risk of death.

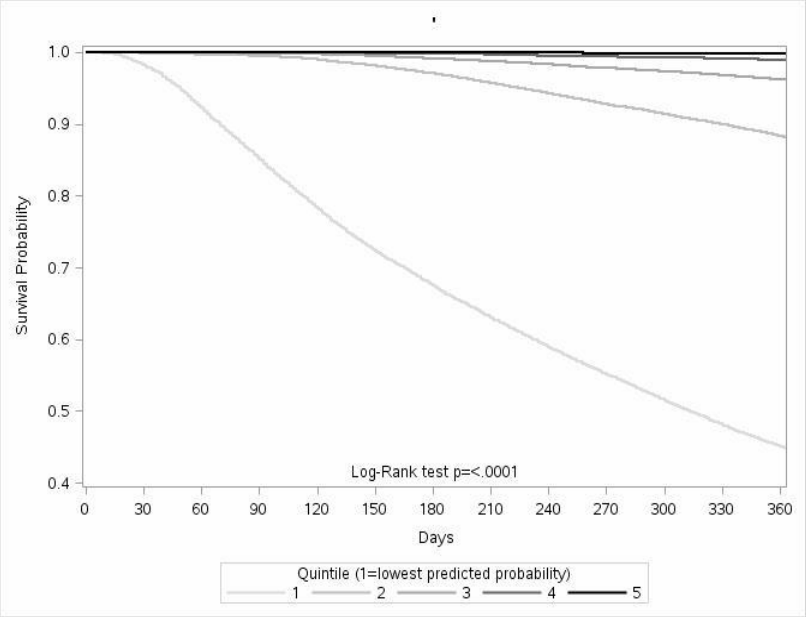

eFigure 2b. Year 1 – Kaplan Meier survival curve when patients grouped by quintiles of 1-year predicted risk of death.

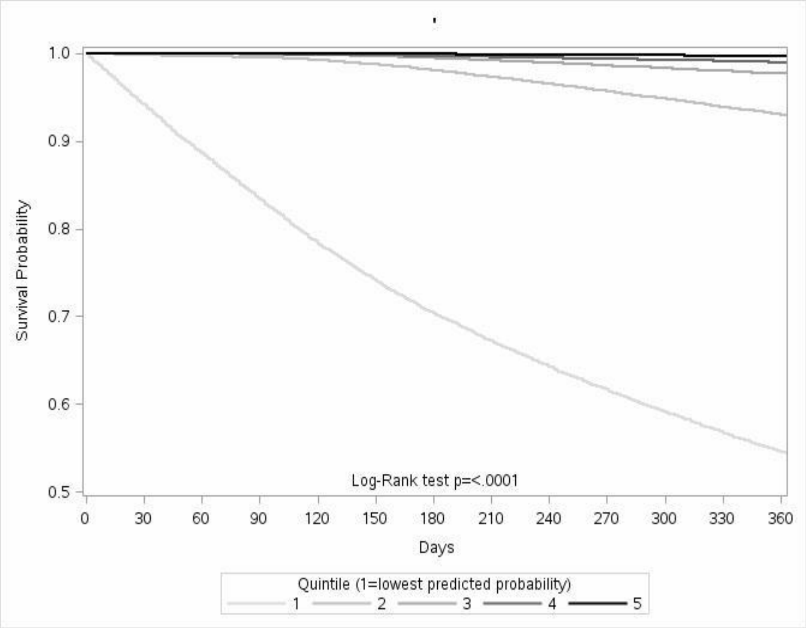

eFigure 2c. Year 2 – Kaplan Meier survival curve when patients grouped by quintiles of 1-year predicted risk of death.

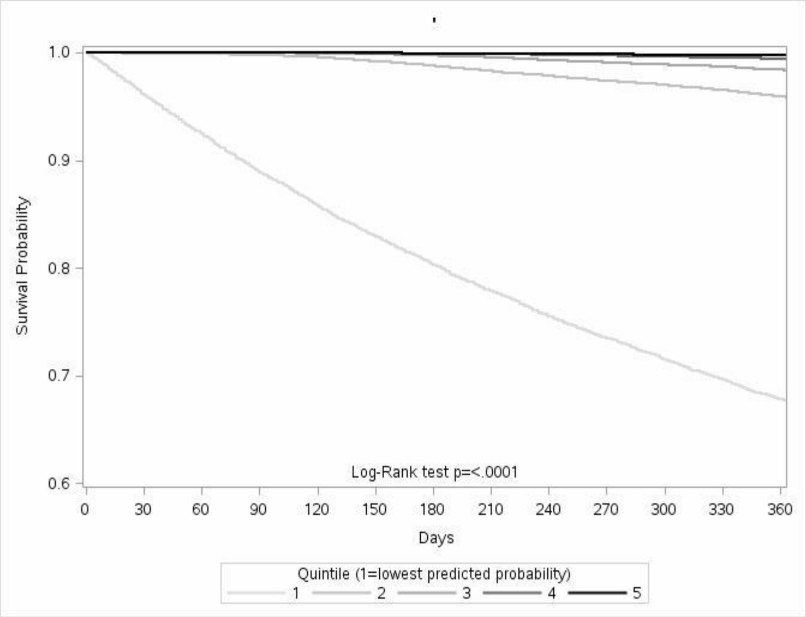

eFigure 2d. Year 3 – Kaplan Meier survival curve when patients grouped by quintiles of 1-year predicted risk of death.

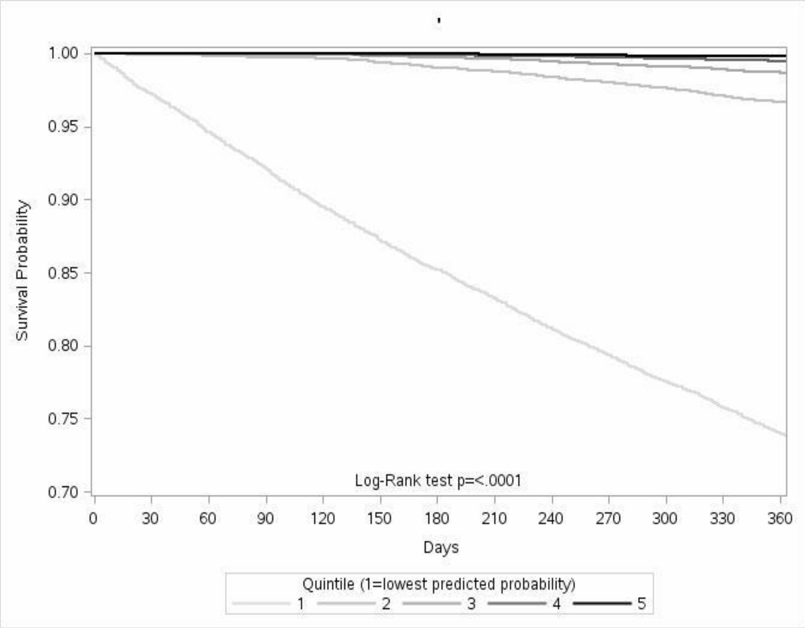

eFigure 2e. Year 4 – Kaplan Meier survival curve when patients grouped by quintiles of 1-year predicted risk of death.

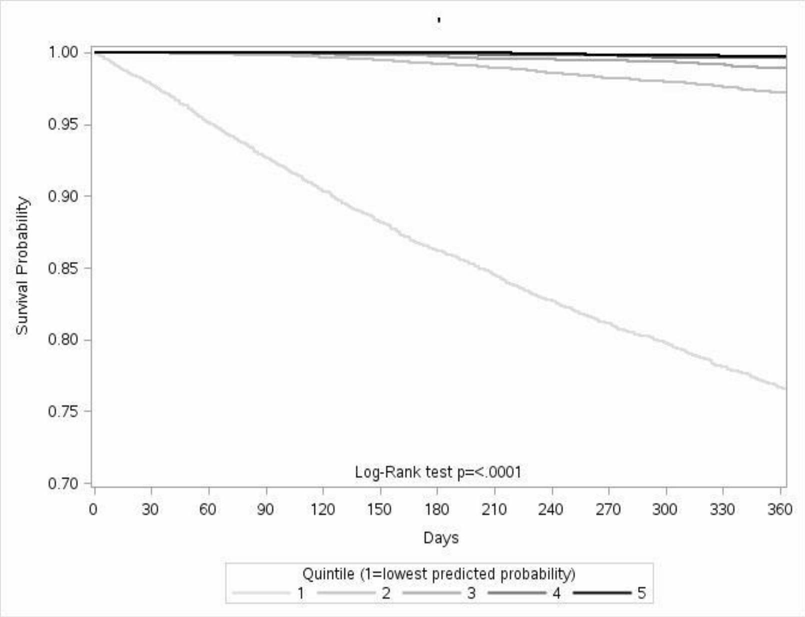

© 2020 Seow H et al. *JAMA Network Open.*
